# Accelerated bottom-up drug design platform enables the discovery of novel stearoyl-CoA desaturase 1 inhibitors for cancer therapy

**DOI:** 10.18632/oncotarget.21545

**Published:** 2017-10-06

**Authors:** Christina A. von Roemeling, Thomas R. Caulfield, Laura Marlow, Ilah Bok, Jiang Wen, James L. Miller, Robert Hughes, Lori Hazlehurst, Anthony B. Pinkerton, Derek C. Radisky, Han W. Tun, Yon Son Betty Kim, Amy L. Lane, John A. Copland

**Affiliations:** ^1^ The Mayo Clinic Graduate School of Biomedical Sciences, Mayo Clinic, Rochester, MN, USA; ^2^ Department of Neuroscience, Mayo Clinic, Jacksonville, FL, USA; ^3^ Department of Cancer Biology, Mayo Clinic, Jacksonville, FL, USA; ^4^ Department of Radiation Oncology, The University of Texas MD Anderson Cancer Center, Houston, TX, USA; ^5^ Department of Chemistry, University of North Florida, Jacksonville, FL, USA; ^6^ Modulation Therapeutics, Inc. Morgantown WV, USA; ^7^ Conrad Prebys Center for Chemical Genomics, Sanford Burnham Medical Discovery Institute, La Jolla, CA, USA; ^8^ Department of Hematology/Oncology, Mayo Clinic, Jacksonville, FL, USA; ^9^ Department of Neurosurgery, Mayo Clinic, Jacksonville, FL, USA

**Keywords:** stearoyl CoA desaturase, lipid metabolism, high throughput drug screening, cancer, drug discovery

## Abstract

Here we present an innovative computational-based drug discovery strategy, coupled with machine-based learning and functional assessment, for the rational design of novel small molecule inhibitors of the lipogenic enzyme stearoyl-CoA desaturase 1 (SCD1). Our methods resulted in the discovery of several unique molecules, of which our lead compound SSI-4 demonstrates potent anti-tumor activity, with an excellent pharmacokinetic and toxicology profile. We improve upon key characteristics, including chemoinformatics and absorption/distribution/metabolism/excretion (ADME) toxicity, while driving the IC50 to 0.6 nM in some instances. This approach to drug design can be executed in smaller research settings, applied to a wealth of other targets, and paves a path forward for bringing small-batch based drug programs into the Clinic.

## INTRODUCTION

Increased fatty acid metabolism is a hallmark of oncogenesis [1-3], and subsequently targeting constituents of lipid biosynthesis is a new focus for developing new anti-cancer therapies. Unlike normal tissues, which rely on exogenous uptake of free fatty acids (FA) from the bloodstream [4], *de novo* lipogenesis contributes a significant portion of the energy requirement needed for tumor growth. Therefore, targeting metabolic enzymes that are critical for cancer cell fatty acid metabolism, but not essential in normal cells, represent a new strategy for cancer therapies. Here, we present a novel computational strategy to aid the synthesis of unique compounds that target stearoyl CoA desaturase 1 (SCD1), a rate-limiting lipogenic enzyme that catalyzes the synthesis of Δ-9 monounsaturated fatty acids (MUFA) oleic acid (OA) and palmitoleic acid (PA)[5]. SCD1 overexpression is observed in a multitude of aggressive malignancies [6-8], and targeted inhibition of this enzyme has been previously shown to impair tumor cell proliferation, and produce tumor-specific cellular stress and apoptosis in representative tumor models [6, 8].

Although different SCD1 inhibitors have been identified using high-throughput screening methods [9, 10], this strategy often relies on structure-based approaches, where both the target and ligand structures need to be present. On the other hand, discovery of SCD1 inhibitors such as MF-438, MK-8245, and SAR707 required the manipulation of the medicinal scaffold of known SCD1 inhibitors [11-13]. In both circumstances, the quality of the final drug is limited by the availability of compound libraries or existing inhibitors. We propose a simple, cost-effective, bottom-up strategy that combines the benefit of having a wealth of ligand information for generating novel compounds, and then screening these compounds in a series of reductive filters using structure-based information, such as, shape, docking, and 3D quantitative structure-activity relationship (QSAR) modeling [14-16]. This approach of virtual exhaustive derivatization followed by functional screening allows for the examination of all structural possibilities to identify novel compounds. Furthermore, results of functional testing can be used to modify the 3D-QSAR in a machine-based learning feedback strategy to more definitively ascertain relevant functional groups necessary for inhibitor function, and improving the selection of second generation inhibitors.

To demonstrate the applicability of our drug development platform, we generated several highly potent, targeted inhibitors of SCD1. Pharmacokinetic analysis of our lead compound, SSI-4, demonstrates excellent oral bioavailability as well as anti-tumor activity when tested in patient-derived xenograft (PDX) models of clear cell renal cell carcinoma (ccRCC). We show that the streamlined process from initial compound design to biological validation can produce unique molecules with desirable pharmacological properties that are not available in existing compounds. This approach to rational drug design thus provides an efficient way to develop new small molecule inhibitors targeting a variety of potential therapeutic targets.

## RESULTS

### Compound library generation

**Figure 1:**
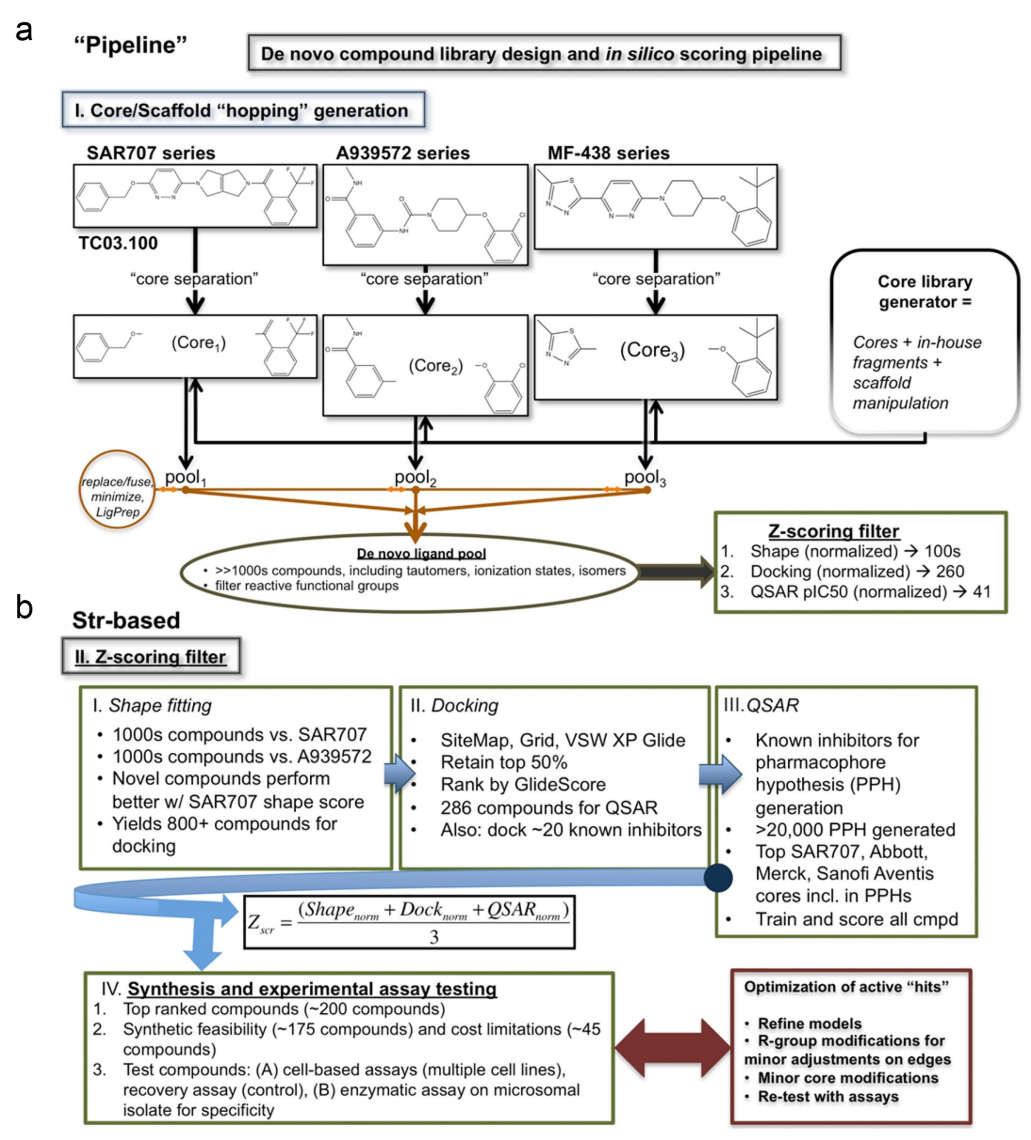
*De novo* compound library design and *in silico* scoring pipeline **a**. Core, or scaffold, hopping generation for three known commercial SCD1 inhibitors (SAR707, A939572, and MF-438) is shown. The central scaffold is separated from the compound (“core separation”) leaving the binding features from the edge of each compound. The core library generator then inserts new cores fuses the edges, minimizes the structure energy, and prepares the ligands (LigPrep). The de novo ligands are then pooled and screened for reactive functional groups. The final set of compounds is then fed into our reductive Z-scoring filter. **b**. Structure-based reductive filter for SCD1 specific compounds. The Z-scoring filter operates in three iterative steps: Shape filter, Docking filter, and QSAR filter. The shape filter generates 100s of conformers for each of the thousands of compounds generated to best fit either SAR707 or A939572. Best fit of compounds with SAR707 had most surviving compounds (>800), thus selected for next filter, docking. Docking filter was applied to >800 compounds from the shape filter. Glide-XP docking retaining top 50% and addition of known inhibitors yields a pool of 286 compounds for QSAR filtering. A QSAR model was made from over 20,000 pharmacophore hypotheses based on a set of 32 known compounds ranging from low nanomolar activity to high milimolar (no activity). The QSAR model trained on this dataset generating a final pool of compounds with good predicted IC50. The algorithm for ranking these final 242 compounds is shown. A subset of 38 compounds from the Z-score filtering was selected for synthesis and testing. Compounds were then tested with cell-based assay and enzymatic assay for activity and specificity. Optimization for these compounds may proceed as needed.

**Figure 2:**
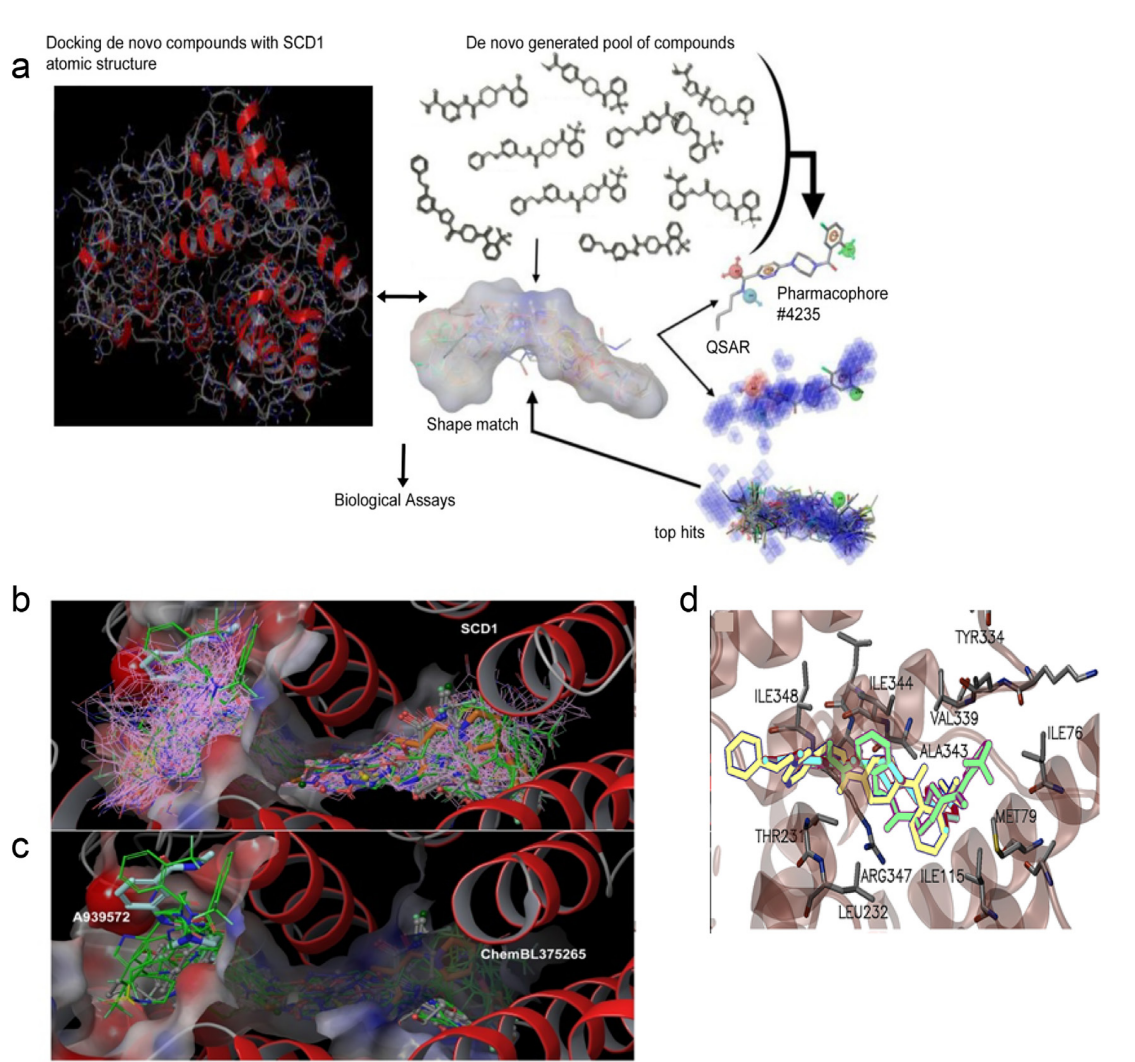
Discovery of SSI-1, SSI-2, SSI-3, and SSI-4 as novel inhibitors of SCD1 (**a**) Schema of *in silico* modeling strategy, where compounds are identified through core, or scaffold-hopping generation, followed by QSAR pharmacophore shape matching after identification of reactive functional groups. Top hits are then validated through functional screening to identify novel lead compounds. (**b**) Docking of novel generated compounds with SCD1 protein is given. The binding pocket is shown with the electrostatic colored surface. Binding poses for all compounds that survived the shape filter to docking filtration. Docking poses for all good scoring compounds (docking score < -8.0) are rendered in pink wire frame. Two known inhibitors are shown in cyan (A939572) and orange (ChemBL375265) CPK rendering. Structures shown in green licorice rendering represent the top 282 compounds from docking. (**c**) Docking of 38 synthesized compounds (green sticks) is shown with two known compounds (A939572 and ChemBL375265). The surface of the binding pocket reveals a very deep tunnel for substrate binding with electrostatic charge distribution shown on the surface. SCD1 helical regions are rendered as red ribbons. (**d**) Ligand interaction map for three top de novo inhibitors is given. Rendered as licorice models, SSI-1, SSI-3, and SSI-4 are shown in yellow, green, and blue. Adjacent residues within 5Å of the inhibitors are rendered in standard color (carbon-gray, oxygen-red, nitrogen-blue) with the alpha-helical region rendered in transparent red ribbons for clarity of view.

To identify a pool of unique compounds, we combined computational-based screening methods, including multiple rounds of filtration with biological analysis to determine candidate functionality (Figure 1, Figure 2a). The *de novo* ligands were first decomposed from A939572, MF-238 and SAR707, which had the cores stripped away and only the periphery/”edges” retained (Figure 1). The deconstructed cores are allowed to sample from a variety of pools to get novel chemical structures that adhere to the driving force of the algorithms employed and subsequently feed into the z-scoring matrix, as described in the Methods. Shape filtering was employed to pare down the database of compounds with poor shape metrics to known inhibitors, which we compared using either A939572 or SAR707 (Supplementary Figure 1a-1b). Each ligand was allowed to generate 100s of conformers for maximal shape overlay between the candidate and existing compounds. Despite the uniqueness of each parent compounds core, the overall best fit was with SAR707 (Figure 2b), which has low nanomolar inhibitory concentration with human liver cell-derived SCD1. Over 800 novel compounds were retained after this initial filtering step, reduced from several 1000’s (Table 1, Supplementary Table 1). Top inhibitor shape scores were 0.513, 0.881, 0.803, 0.660, and 0.642, for SSI-1, SSI-2, SSI-3 and SSI-4, respectively (Table 2).

**Table 1:** Comparison of QSAR results with experimental findings for activity.

Ligand Name	Rank 1 pIC50	Single Pt Act (10μM)	Known IC50 (nM)	pIC50 (nM)	<500 nM?	MW
DaiichiSankyo[50] 3nM	1	NA	3	1.87	Y	452.45
CVT-12-012 36nM	2	NA	36	13.52	Y	434.42
SAR707[12] 8nM	3	NA	8	14.49	Y	466.46
TC03.10	4	No		30.98	Y	537.58
TC03.48	5	No		40.83	Y	538.57
Abbott 10b[51]	6	NA	58	52.61	Y	464.32
Abbott 7c[51]	7	NA	51	67.77	Y	419.87
TC03.1	8	Not tested		70.96	Y	469.46
TC03.100 Blind Control 10-40 nM SAR707	9	NA		72.62	Y	418.90
TC03.18	10	No		72.62	Y	519.54
Abbott 10g[51]	11	NA	100	91.42	Y	436.44
SetA.70	12	No		91.42	Y	408.90
TC03.4	13	Y		104.71	N	456.46
TC03.14	14	No		109.91	Y	483.49
SSI-3	15	Y		126.19	Y	498.50
TC03.6	16	No		129.13	Y	495.50
TC03.37	17	Y		155.25	Y	539.56
Analog3[52]	18	NA	107	170.23	Y	443.43
SetB.74	19	No		170.23	Y	420.43
DaiichiSankyo[53]	20	NA	100	178.25	Y	514.63
Xenon LCF369	21	NA	120	182.40	Y	465.45
TC03.61	22	Y		191.00	Y	510.56
TC03.8	23	No		219.30	Y	523.55
SetA.69	24	Y		224.40	Y	394.87
MerckFrost MF-152	25	NA	100	240.45	Y	384.38
TC03.15	26	Y		246.05	Y	483.49
SetB.68	27	No		282.51	Y	392.38
SetB.73	28	No		289.09	Y	406.40
SetA.61	29	No		302.71	Y	395.86
TC03.46	30	No		302.71	Y	483.49
SetB.70	31	No		331.92	Y	407.39
SSI-4	32	Y	7	339.65	Y	388.85
A939572 37nM	33	NA	37	372.42	Y	387.87
SSI-2	34	Y		381.09	Y	484.48
Abbott 10e[51]	35	NA	470	447.74	Y	405.40
TC03.5	36	Y		468.85	Y	470.49
TC03.66	37	Y		468.85	Y	523.55
TC03.23	38	Y		479.77	Y	526.51
SetB.52	39	No		479.77	Y	473.49
SetB.61	40	No		550.85	N	484.48
TC03.31	41	No		632.46	N	497.52
SetB.66	42	No		709.63	N	406.40
TC03.53	43	No		726.16	N	524.54
Abbott[54]	44	NA	400	833.74	N	353.42
TC03.47	45	No		853.16	N	533.56
SetA.100 Blind Control 400 nM (28c, Abbott ChemBL375265)	46	NA	400	873.03	N	389.84
SetB.49	47	No		957.26	N	469.48
Abbott 10a[51]	48	NA	6000	1099.08	N	385.42
SSI-1	49	Y		1099.08	N	449.43
TC03.56	50	N		1177.69	N	483.49
SetA.68	51	Y (no rescue)		1321.39	N	397.88
TC03.41	52	Y		1352.17	N	497.52
SetA.1	53	No		1482.62	N	385.85
SetB.69	54	No		1482.62	N	420.43
SetB.34	55	No		2760.77	N	392.38
Abbott 10h[51]	56	NA	10000	5508.46	N	397.43
SetB.75	57	No		5902.42	N	463.46
Analog2[52]	58	NA	2680	49094.18	N	381.35
Analog1[52]	59	NA	10000000	166352.75	N	367.33

**Table 2:** Composite Z-score for modeling of all de novo ligands designed using Docking, Shape, and QSAR for ligand-guided predictions of high affinity drug-like compounds.

	Drug candidate		Sub-metrics		ComboScore	
**RANK**	**NAME**	**docking (normalized)**	**Shape Sim (normalized)**	**QSAR (normalized)**	**Z-score sub =Di+Si+Qi**	**Avg Z-score**
1	SSI-2‡	0.99	0.77	0.90	2.67	0.89
2	TC03.18	0.91	0.77	0.96	2.64	0.88
3	SSI-3‡	0.88	0.81	0.94	2.62	0.87
4	SAR707 8nM (TC03.100)	0.63	1.00	0.96	2.59	0.86
5	TC03.14	0.84	0.79	0.95	2.58	0.86
6	TC03.46	0.92	0.81	0.85	2.58	0.86
7	TC03.10	0.84	0.74	0.98	2.56	0.85
8	TC03.37	0.85	0.71	0.92	2.48	0.83
9	TC03.1	0.78	0.71	0.96	2.45	0.82
10	TC03.6	0.73	0.76	0.94	2.42	0.81
11	TC03.23	0.90	0.72	0.76	2.37	0.79
12	TC03.48	0.57	0.80	0.98	2.34	0.78
13	TC03.61	0.72	0.80	0.81	2.33	0.78
14	TC03.31	0.91	0.71	0.68	2.31	0.77
15	TC03.5	0.84	0.70	0.77	2.30	0.77
16	SetB.74	0.78	0.57	0.91	2.27	0.76
17	Abbott 10g phenoxyl 100nM	0.77	0.54	0.95	2.26	0.75
18	TC03.15	0.63	0.74	0.88	2.25	0.75
19	Abbott 7c 2chlorophenoxyl 51nM	0.85	0.43	0.97	2.24	0.75
20	SSI-4‡	0.78	0.62	0.83	2.23	0.74
21	SetB.73	0.78	0.60	0.86	2.23	0.74
22	DaiichiSankyo 3nM [50]	0.67	0.64	0.91	2.23	0.74
23	SetB.7	0.77	0.58	0.88	2.23	0.74
24	TC03.53	0.84	0.71	0.64	2.19	0.73
25	Merck Inhibitor <100 nM	0.70	0.47	1.00	2.16	0.72
26	TC03.47	0.89	0.68	0.57	2.15	0.72
27	SetB.61	0.69	0.62	0.82	2.13	0.71
28	TC03.7	0.80	0.73	0.55	2.08	0.69
29	TC03.8	0.44	0.73	0.89	2.07	0.69
30	SetA.69	0.74	0.42	0.89	2.05	0.68
31	SetB.6	0.68	0.58	0.74	2.00	0.67
32	SetB.52	0.57	0.64	0.76	1.96	0.65
33	SetB.66	0.71	0.60	0.65	1.95	0.65
34	TC03.66	0.52	0.65	0.77	1.93	0.64
35	TC03.56	0.86	0.65	0.41	1.92	0.64
36	SetA.61	0.67	0.40	0.85	1.92	0.64
37	SetA.70	0.55	0.39	0.95	1.89	0.63
38	SetB.69	0.82	0.62	0.26	1.70	0.57
39	SetA.100 control (400nM)	0.72	0.40	0.56	1.68	0.56
40	SetB.49	0.57	0.57	0.52	1.67	0.56
41	SSI-1‡	0.60	0.61	0.45	1.66	0.55
42	TC03.41	0.57	0.72	0.32	1.62	0.54
43	SetA.68	0.74	0.38	0.34	1.46	0.49
44	SetA.59	0.72	0.40	0.31	1.42	0.47
45	DaiichiSankyo IC50 100 nM[53]	0.87	0.52	0.00	1.39	0.46
46	Abbott 10d phenoxyl 51nM	0.85	0.45	0.00	1.30	0.43
47	Abbott 10c phenoxyl 38nM	0.81	0.42	0.00	1.23	0.41
48	SetA.1	0.54	0.39	0.26	1.19	0.40
49	SetB.34	0.80	0.56	-0.38	0.98	0.33
50	SetB.75	0.57	0.58	-1.05	0.09	0.03

### Candidate inhibitors have excellent predicted binding affinity for SCD1

In order to estimate the affinity of the compounds for SCD1 binding region as compared with that of over 20 known inhibitors, including A939572, SAR707, and MF-438, docking with over 500 top generated compounds was performed using the “Scaffold/Core Hopping” technology [17-22]. The binding pocket for SCD1 is a long funnel-shape (Figure 2b), which can easily accommodate stearoyl-CoA. Using the Virtual Screening Workflow (VSW) docking process, we proceeded through the highest level of Glide precision, XP level docking [23, 24]. Also implementations of novel conformational sampling algorithms were utilized; our methods have been previously described [25-29]. As shown in Figure 2b-c, the region of SCD1 is highly alpha-helical in nature and has indicated electrostatic distribution for lipid binding. In order to illustrate candidate inhibitor docking poses (pink wire frame) throughout the identified binding pocket of SCD1, we overlaid their distribution with all commercial inhibitors (licorice style CPK molecules), which include A939572, GSK993, MF-438, ChemBL375265, and SAR707 (Figure 1b). Also included in the overlay are the 41 compounds we had synthesized (green stick rendering) (Figure 2b). To better illustrate the position of the 41 synthesized compounds relative to the known inhibitors A939572 (cyan) (human IC_50_=37 nM) and ChemBL375265 (orange) (human IC_50_ =400 nM), we showed the docking poses of this group independently (Figure 2c). ChemBL375265 binds deep at the base of the pocket, whilst A939572 is seen closer to the opening of the binding pocket (Figure 2c). We predict that relative binding position of the inhibitors may alter substrate kinetics, thus affecting inhibitory concentration needed.

To identify which structural components of our experimental inhibitors are important for protein binding to SCD1, we interrogated SCD1 amino acid R-group interactions with our active designer small molecules SSI-1, SSI-2, SSI-3, and SSI-4. Within the tunnel-like crevice of the enzyme where each of these inhibitors fit, Met79, Val339, Ile115, Thr231, Leu232, Tyr334, Ala343, Arg347, and Ile348 all approach within 4.5 Å of these inhibitors (Figure 2d). Additionally, we calculate the nitro-groups from all 4 top hits participate in electrostatic interactions with the Arg residues, and transient π-cloud interactions occur with the phenyl-substituted rings from SSI-3 (Figure 2d). Further stabilizing interactions likely occur between the hydrophobic residues and aliphatic atoms from all four top hits (Figure 2d).

The outcome of our docking analyses is a set of 286 compounds with docking scores ranging from -12.55 kca/mol to -7 kcal/mol, including our top scoring ligands SSI-1 (-9.92 kcal/mol), SSI-2 (-9.0 kcal/mol), SSI-3 (-10.38 kcal/mol), and SSI-4 (-10.91 kcal/mol) (Supplementary Table 2). Previous docking-only based compound screening fell short of our desired screening and design expectations (data not shown), which encouraged our use of a Z-scoring method for combining Shape and QSAR with Docking into a rubric for synthesis selection to predict inhibitory concentrations of candidate small molecules.

### Predicted SCD1-inhibitory activity of lead compounds

**Figure 3:**
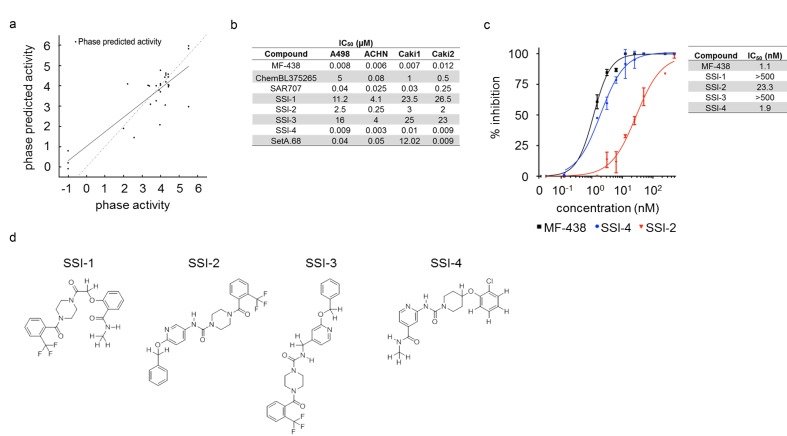
Inhibitory activity of SSI-1, SSI-2, SSI-3, and SSI-4 (**a**) Result of QSAR model for the top pharmacophore hypothesis is shown with the linear correlation of phase predicted activity versus known activity for commercially available compounds that range in low activity to high activity level. (**b**) IC_50_ values of known and novel SCD1 inhibitors generated using linear regression modeling. (**c**) Summary of enzymatic dose-response curves for known and experimental SCD1 inhibitors generated by in vitro SCD1 enzymatic inhibition assays as determined by LC/MS. Error bars indicate standard deviation for n=2 replicates. (**d**) The chemical structures of SSI-1, SSI-2, SSI-3, and SSI-4 are shown.

To further refine the selection of inhibitors and identify appropriate candidates for synthesis, the top 286 compounds from docking were fed into the QSAR modeling program within 3D-QSAR Schrödinger program [30, 31]. Here the QSAR is built on pharmacophore hypotheses (PPHs) generated for a wide range of IC_50_ values published for chemical inhibitors of SCD1 (2 nM to >10,000 nM). In total we took 32 compounds over this range to generate a training set and create >20,000 pharmacophore models. Each known compound was then fitted to the best PPH that fit all the data (Supplementary Figure S1c-e). As an example, the aromatic ring structures of our lead compound SSI-4 align more closely with the predicted PPH model AAAHHR.4452 (where spatial orientation of the aromatic ring structures as predicted by the PPH model are shown as orange rings) (Supplementary Figure 1f) as compared to A939572, SAR707, and ChemBL375265 (Supplementary Figures S1c-e). Using all of these PPHs, we were able to establish a good activity to predicted activity linearity (Figure 3a). Then, the top 286 compounds were tested with the QSAR filter. The output of the 3D-QSAR measurements gave predicted IC_50_’s for all tested compounds, determining approximately 140 compounds worth testing (Table 1, Supplementary Table 3).

From these 140 compounds, the top 45 were selected through a second Z-filter comprised by combined normalized scoring. The final Z-score for each compound was determined as: (1) $Z_{scr}=\frac{\left({Shape}_{norm}+{Dock}_{norm}+{QSAR}_{norm}\right)}{3}$, which takes the average of the sums of the Shape scores that were normalized to the top performing compound (*Shape*_*norm*_), the Dock scores that were normalized with the best docking compound (-12.55 kca/mol) (*Dock*_*norm*_), and the 3D-QSAR normalized to the best predicted IC_50_ value (*QSARnorm*). The top 40-45 compounds were selected for synthesis and testing, from the 140 compounds that entered the Z-scoring matrix (Table 2). Additionally, we performed a chemoinformatics assessment for all *de novo* compounds to determine whether the compound was ‘drug-like’ or ‘lead-like’ in terms of violations of Lipinski or Jorgensen Rules before proceeding (Table 3, Supplementary Table 4).

**Table 3:** SCD1 inhibitor’s “druglikeness” for each candidate based # violations of to Lipinski’s Rule of 5 and Jorgensen’s Rule of 3.

Drug candidate Compound	Rule of 5	Rule of 3
TC03.1	1	1
TC03.10	2	1
TC03.14	1	1
TC03.15	1	1
SSI-2‡	1	1
TC03.18	2	1
TC03.23	1	1
TC03.31	1	1
TC03.32	1	1
TC03.33	2	1
TC03.37	1	1
TC03.4	1	1
TC03.41	1	1
SSI-3‡	1	1
TC03.46	1	1
TC03.47	1	1
TC03.48	2	1
TC03.5	1	1
TC03.53	2	1
TC03.56	1	1
TC03.6	1	1
TC03.60	1	1
TC03.61	1	1
TC03.66	2	1
TC03.8	2	1
SetA.100 control	0	1
SSI-4‡	0	0
SetA.59	0	0
SetA.61	0	0
SetA.68	0	0
SetA.69	0	0
SetA.70	0	0
SSI-1‡	0	1
SetB.34	0	1
SetB.49	0	0
SetB.52	0	1
SetB.6	0	1
SetB.61	0	1
SetB.66	0	1
SetB.69	0	1
SetB.7	0	1
SetB.73	0	1
SetB.74	0	0
SetB.75	0	0

### SSI-(1-4) are novel inhibitors of SCD1 enzymatic activity

After synthesis feasibility and cost analysis, we synthesized 38 of the selected compounds. Utilizing a high-throughput proliferative screen with 4 ccRCC cell lines, we identified 5 compounds that produced a >40% decrease in proliferation in at least 2/4 cell lines: SSI-1, SSI-2, SSI-3, SSI-4, and SetA.68 (-9.34 kcal/mol) (Supplementary Figure 2a). Using linear regression modeling generated from dose-response curves, proliferative inhibitory concentrations (IC_50_) of these 5 compounds were established (Figure 3b, Supplementary Figure 2b-2f). To evaluate inhibition of SCD1 by these molecules, we used LC/MS to quantify changes in SCD1-catalyzed conversion of stearoyl-CoA to oleoyl-CoA in response to drug treatments *in vitro* using SCD1 protein extracted from murine liver (Figure 3c, Supplementary Figure 2g). SetA.68 did not inhibit oleoyl-CoA conversion, and was excluded from further analysis. Of note, the experimental IC_50_ determined for MF-438 (1.1 nM), included as a positive control, corresponds well with the published IC_50_ (2.3 nM) [11]. The chemical structures for the novel SCD1 inhibitors SSI-1, SSI-2, SSI-3, and SSI-4 are shown in Figure 3d. Synthesis routes and spectral data including NMR and MS are also provided (Supplementary Figures 3-6).

### SSI-(1-4) reproduce known biological stress responses in tumor cells

**Figure 4:**
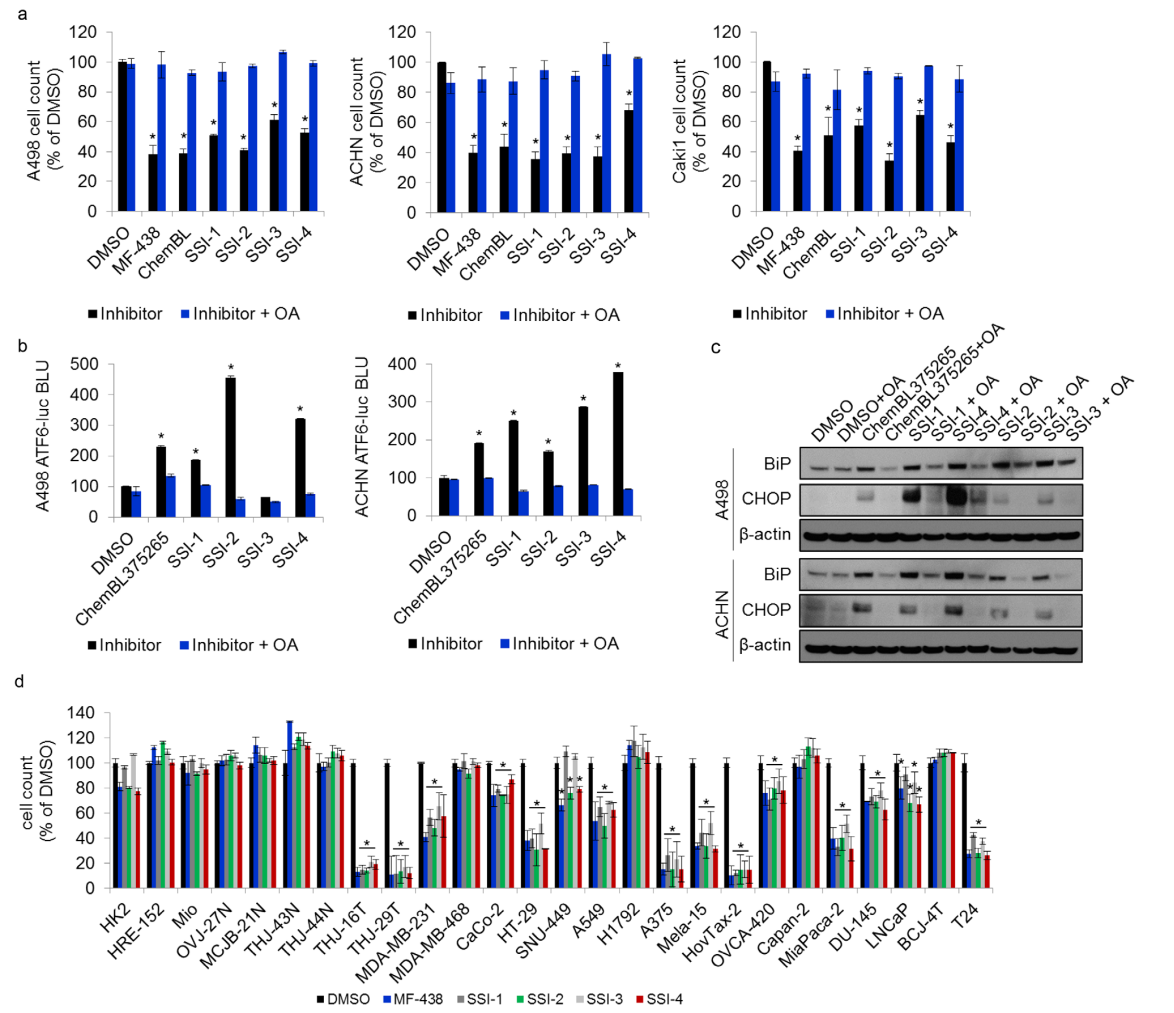
Experimental SCD1 inhibitors induce tumor endoplasmic reticulum stress (**a**) Results of 72 hour proliferation assay in 3 ccRCC cell lines treated with the IC_50_ of known or experimental SCD1 inhibitors, along with adjuvant exogenous oleic acid (5µg/mL). (**b**) ATF6-UPRE luciferase reporter assay of A498 and ACHN cells treated with the IC_50_ dose of ChemBL375265 or SSI-(1-4), +/- OA supplementation (5µg/mL). Results are presented as relative bioluminescence (BLI). (**c**) Western blot for protein expression of UPR markers BiP and CHOP in A498 and ACHN cells treated with the IC_50_ doses of ChemBL375265, and SSI-(1-4) +/- OA supplementation (5µg/mL). Beta-actin is used as a loading control. (**d**) Proliferative response in patient-derived normal cells, and established tumor cell lines to treatment with MF-438 or SSI-(1-4).

To further confirm SCD1 target specificity for SSI-(1-4), we repeated the proliferative challenge in RCC cell lines in the presence of exogenous oleic acid (OA), which demonstrates rescue of the cytotoxic defects induced by SCD1 inhibitors [6]. OA fully restored proliferation in all cells treated with proliferative IC_50_ dose of MF-438 and ChemBL375265 controls, as well as SSI-(1-4) (Figure 4a). Induction of the unfolded protein response (UPR) is a known biological response to SCD1 inhibition in tumor cells [6, 8]. SSI-(1-4) were each able to significantly induce luciferase activity in either A498 or ACHN cells expressing an ATF6-UPRE luciferase-reporter (Figure 4b), where activating transcription factor 6 (ATF6) is a key regulator of the UPR [32]. The addition of exogenous OA was sufficient to reverse the activation of ATF6 (Figure 4b). Moreover, exogenous OA inhibited SSI-(1-4) mediated upregulation of the UPR markers BiP (heat shock 70 kDa protein) and CHOP (damage inducible transcript 3) protein levels in both A498 and ACHN cells (Figure 4c). Collectively these findings confirm that SSI-(1-4) reliably recapitulates the biological responses observed in tumor cells treated with known SCD1 inhibitors. Given that SSI-4 exhibited the most potent enzymatic inhibitory efficacy against SCD1 (Figure 3b-3c), we performed a full kinome scan to test for potential off-target effects *in vitro* via non-specific binding to 468 known kinases. Binding to just 1 of 403 non-mutant kinases (CDKL2) was observed with SSI-4 at a concentration of 100 nM, and none at 10 nM. Thus, SSI-4 is not predicted to have any major off-target effects through kinase inhibition (Supplementary Figures 7 and 8).

**Figure 5:**
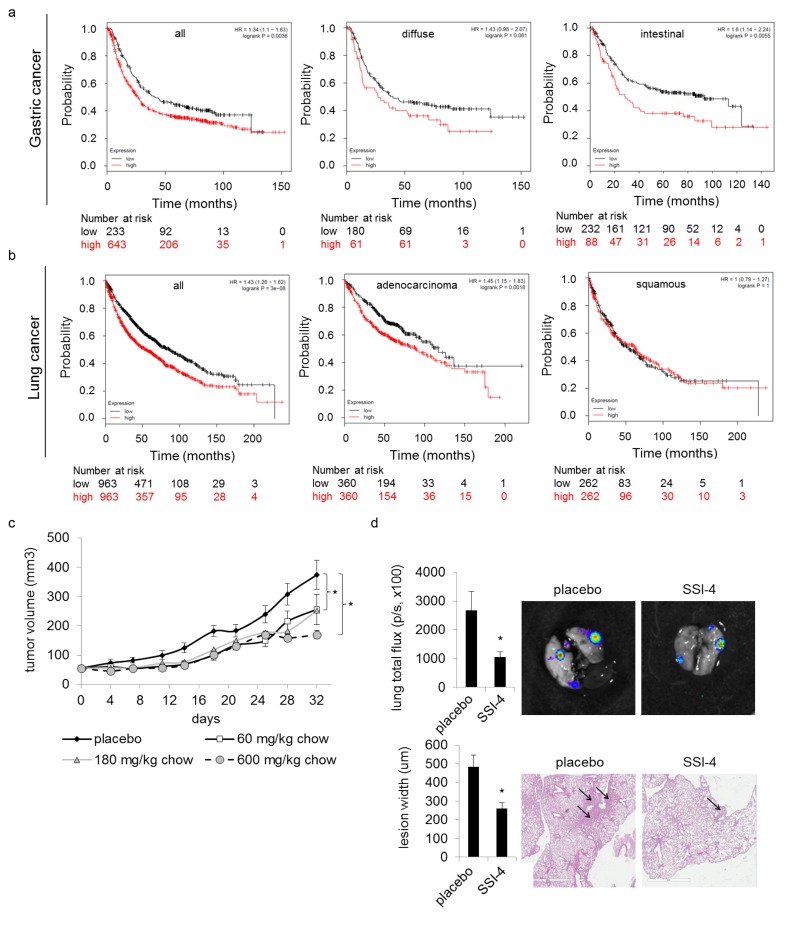
SSI-4 attenuates tumor progression Kaplan Meier survival analysis of indicated subtypes of (**a**) gastric and (**b**) lung cancer patients sorted into high versus low SCD1 mRNA expression, for multiple disease subtypes. Patient risk assessment at indicated mean follow-up is shown. (**c**) Tumor volume (mm^3^) in athymic nude mice bearing subcutaneous A498 xenograft doses with SSI-4 (n=10 per group). (h) Tumor burden of ACHN pulmonary metastasis in response to SSI-4 treatment (180mg/kg) measured by *ex vivo* bioluminescent flux, given in photons/second (n=5 per group). (**d**) Tumor burden of ACHN pulmonary metastasis as determined by H&E staining of lungs from (h).

SCD1 expression correlates with poor cancer patient outcomes as evidenced by decreased survival in multiple cancers including gastric, lung, ovarian and renal cell carcinoma (Figure 5a-5b, Supplementary Figure 9a-9b). We found that SSI-(1-4) impaired tumor cell proliferation in 15/19 different tumor cell lines representing a broad spectrum of cancers, while normal cells remained unaffected (Figure 4d). The inhibitors showed no activity against H1792 cells, which do not express SCD1 (Supplementary Figure 9a). However, for tumor cells that express SCD1, the anti-proliferative effects of SSI-(1-4) did not necessarily correlate with its expression levels (Figure 4d, Supplementary Figure 9c). This suggests that while SSI-(1-4) require the presence of SCD1 to produce anti-tumor effects, their potencies are likely dependent on multiple factors beyond the simple expression levels of the enzyme, such as the presence of compensatory mechanisms for fatty acid metabolism [33]. Our findings in thyroid carcinoma similarly present discordancy between tumor response to SCD1 inhibitors and SCD1 expression [7]. SSI-4 was tested *in vivo* for anti-tumorigenic activity. Oral administration of SSI-4 resulted in growth inhibition of A498 ccRCC tumors (Figure 5c). SSI-4 treatment also reduced pulmonary metastasis formation of ACHN ccRCC (Figure 5d). These data support the notion that SCD1 represents an actionable target for the treatment of metastatic ccRCC.

### SSI-4 exhibits favorable pharmacokinetic and toxicological profile

**Figure 6:**
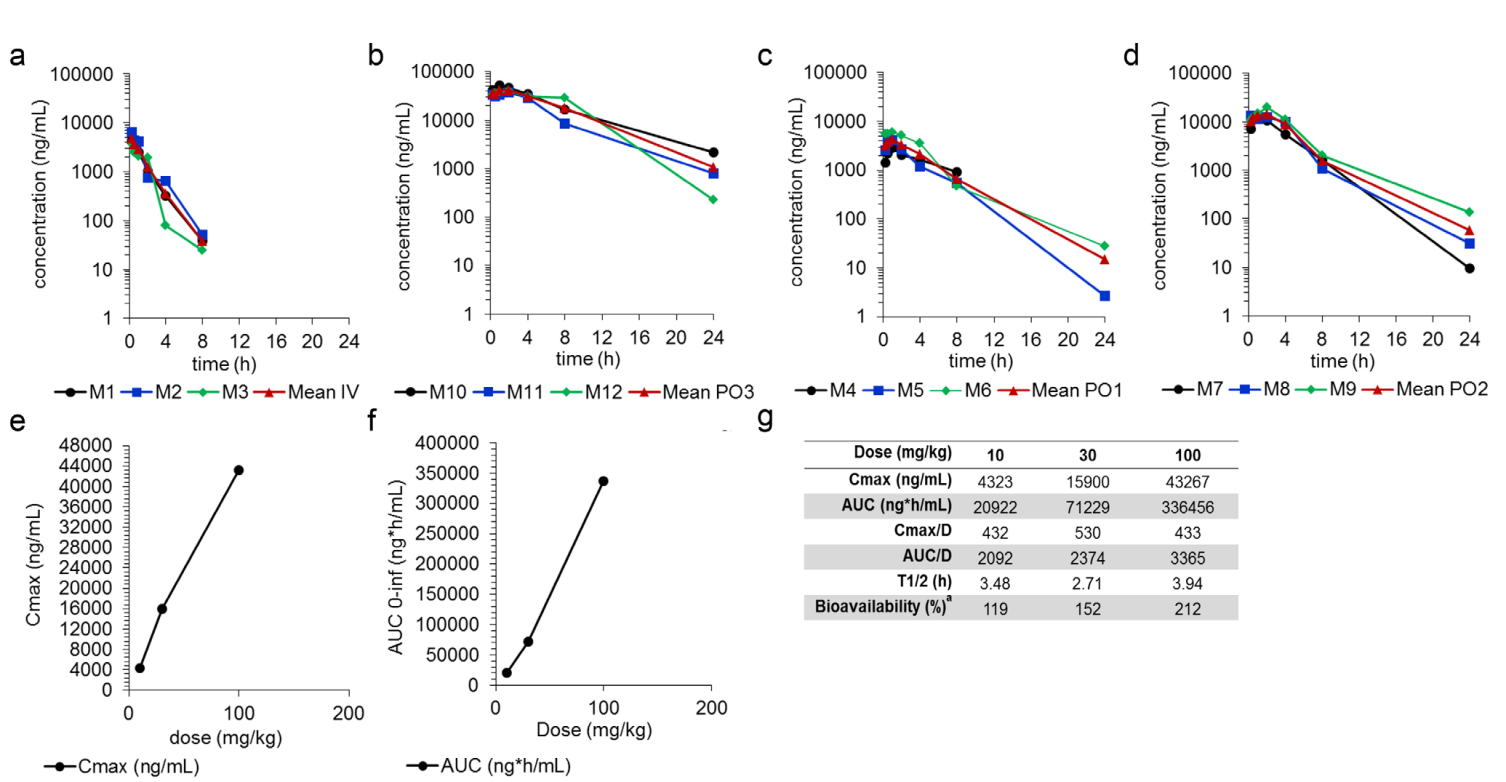
SSI-4 demonstrates excellent bioavailability Serum concentration at different time points after drug delivery following (**a**) IV or (**b**-**d**) oral administration, at indicated concentrations. (**e**) Maximum serum concentration (Cmax) of SSI-4 before the administration of a second dose. (**f**) AUC curve to infinite time. (**g**) Summary of pharmacokinetics for SSI-4, where absolute bioavailability (%) is quantitated with AUC_0-last_ and nominal dose.

To assess whether SSI-4 possesses a good pharmacokinetic profile, we measured the serum concentration of the compound after oral (PO) and intravenous (IV) administration in rat. We observed high levels of SSI-4 in serum after oral administration (Figure 6a-6d) of approximately 4,323ng/mL, 15,900ng/mL, and 43,267ng/mL after 10mg/kg, 30mg/kg, and 100mg/kg doses, respectively (Figure 6e). The median serum terminal half-life (T1/2) for each dose ranged from 2.71-3.94 hours (Figure 6g). Absolute bioavailability after oral administration was determined as 119%, 152%, and 212% for 10, 30, and 100mg/kg, respectively (Figure 6f-g). These data support that SSI-4 demonstrates a favorable drug-like profile, with excellent oral absorption.

**Figure 7:**
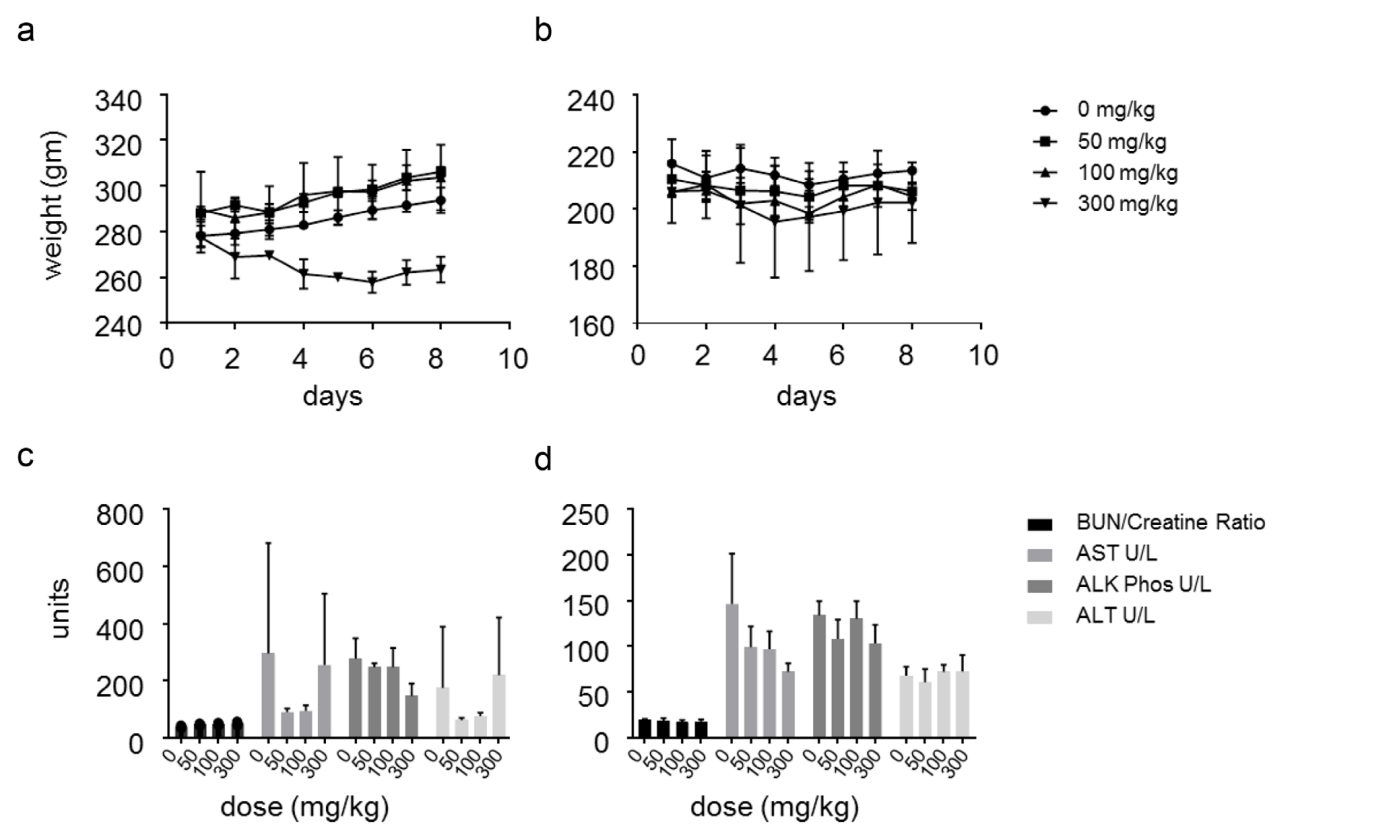
SSI-4 has a favorable toxicity profile (**a**-**b**) Weight changes in male or female Sprague Dawley rats treated daily with indicated doses of SSI-4 for seven days (n=3 per group). (**c**-**d**) Serum profile of indicated enzymes measured on day 8 in male or female Sprague Dawley rats treated daily with indicated doses of SSI-4 for seven days (n=3 per group). (BUN= blood urea nitrogen; AST= aspartate aminotransferase; ALT= alanine aminotransferase; ALK/Phos= alkaline phosphatase).

Toxicological study demonstrated minimal changes in body weight (<10%) in male mice treated daily with oral dose of 300mg/kg of SSI-4 for one week, while no significant changes were observed in females at all doses tested (Figure 7a-7b). No abnormalities in liver enzymes were found at all doses tested in both males and females (Figure 7c-7d). Chronic treatment at the highest dose in tumor-bearing animals appeared to be well tolerated, with ‹10% reduction in body mass even at the highest dose of 600mg/kg after 4.5 weeks of daily treatment (Supplementary Figure S9d). Ocular changes including bilateral squinting were observed as the primary adverse events, and these were reversible upon discontinuation of therapy.

## DISCUSSION

An increasing trend for the use of computational, structure-based studies for accelerating drug discovery and leapfrogging multiple chemical syntheses, with guided 3D-QSAR, or Field-based QSAR, have improved medicinal chemistry studies for drugs [12, 14, 30, 34-40] such as MK-8931 or SAR707. From virtual screening to *de novo* design, evaluation of drug-likeness, and further optimization of drug candidate for improved Pk/Pd and oral bioavailability, in silico methods have been useful tools for speeding results and saving research dollars[14, 40]. The role of computer-aided drug discovery includes both structure-based and ligand-based methods. For structure-based approaches, which in comparison to high-throughput screening, both the target and ligand structure are present, while for ligand-based only the examination of the ligand’s information is used for predicting activity.

Here, our method utilizes both existing ligand information in combination with predictive structure modeling, emancipating the limitations for the high-throughput screen to available drug libraries. We create unique structure-based reductive filters that allow us to better predict inhibitory efficacy as well as drug-likeness, improving the probability of successfully identifying novel targeted inhibitors. The results of functional testing, such as experimental IC_50_, can be deposited within the QSAR modeling to modify screening parameters in a machine-learning based feedback mechanism. This will improve the PPHs used to score candidate inhibitors, enhancing the selection of further generations of inhibitors and potentially a rapid and cost effective strategy for developing new classes of molecules.

Aberrant SCD1 expression is commonly observed in aggressive malignancies, and negatively correlates with patient outcomes, generating a clear prospective for targeted therapy of this enzyme. Using SCD1 inhibitors as our model for drug development, our method allowed successful identification of four unique small molecules, SSI-1, SSI-2, SSI-3, and SSI-4, whose biological efficacy rivals that of leading pharmaceutical grade competitors. Each of these represents novel compounds with new scaffolds and formally unique structures that have not been previously synthesized or tested. This method has broad applicability as it may be used for the discovery of new classes of inhibitors, improving synthesis efficiency, or to strategically manipulate drug analogs that are essential for the generation of optimized variants. Furthermore, it is feasible to execute in smaller research settings that may not have access to existing compound libraries or high-throughput screening tools.

## MATERIALS AND METHODS

### Compounds

A939572 was purchased from BioFine International. MF-438 (2-methyl-5-(6-(4-(2-(trifluoromethyl)phenoxy)piperidin-1-yl)pyridazin-3-yl)-1,3,4-thiadiazole) was synthesized by the medicinal chemistry group at Sanford-Burnham according to the published procedure, and was determined to by >95% pure by LC-MS. All experimental compounds, as well as ChemBL375285, were synthesized by Enamine. All compounds were validated with LCMS and/or ^1^H NMR to meet the minimum requirement of 90% purity.

### Tissues and cell lines

All patient tissue used throughout the course of this study were procured from de-identified patients. This study has been approved by the Mayo Institutional Review Board. Human cell lines including: A498, ACHN, Caki1, Caki2, MDA-MB-231, CaCo-2, HT-29, SNU-449, A549, A375, MiaPaca2, DU-145, LNCaP, and T24 were purchased from the American Type Culture Collection (ATCC). HovTax2, THJ16T, THJ29T, and Mela15 were established in the Copland laboratory. OVCA420, was a gift from Dr. Robert C. Bast Jr. (University of Texas MD Anderson Cancer Center). All mortal normal cells were derived from primary patient tissue. Thyroid cells were grown in RPMI (Cellgro) containing 5% FBS (Hyclone) and 1x penicillin-streptomycin (Invitrogen), at 37° C in humidified conditions with 5% CO_2_. All other cells were cultured in DMEM medium (Cellgro) containing 5% FBS (Hyclone) and 1x penicillin-streptomycin (Invitrogen), at 37° C in humidified conditions with 5% CO_2_.

### DNA isolation and STR analysis

Genomic DNA was extracted from previously established cell lines (MDA-MB-231, CaCo2, HT-29, SNU-449, A549, A375, HovTax2, OVCA420, MiaPaca2, DU-145, LNCAP, T24), and cell lines established in the Copland laboratory (THJ16T, THJ29T, and Mela15) using Purelink™ Genomic DNA mini kit (Invitrogen). Sixteen STR markers were PCR amplified using fluorescently labeled primers from ABI (Applied Biosystems), and were analyzed using ABI 3130 (Applied Biosystems). Peak sizes were calculated versus a co-injected size standard using Gene Marker (Soft Genetics), performed by the Mayo Medical Genome Facility Genotyping Core.

### Growth assays

Cells were seeded at 5,000 cells/well in clear-bottom 96-well plates in triplicate. Drug treatment was applied at 1:1000 in reduced serum conditions (3%). After 72 hours, cells were washed with PBS, and stored at -80^o^C prior to analysis using CyQuant® Proliferation Analysis Kit (Invitrogen) er manufacturers’ protocol for relative fluorescence units. Alternatively, cells were plated 2x10^5^/well in 12-well plates (Genesee Scientific) in triplicate prior to drug treatment. After 120-hour treatment, cell number was established using a Coulter Particle Counter (Beckman). Oleic acid-albumin (Sigma Aldrich) was added to media at 5µM, and was applied adjuvant to drug treatment. Drug stocks were prepared in DMSO (Sigma) at 1000x. IC_50_ dosing per cell line was calculated using CalcuSyn analytical software.

### Luciferase assay

A498, Caki1, and ACHN cells were transiently transfected with p5xATF6-GL3 UPR luciferase reporter (Addgene plasmid#11976) and pRL-CMV-renilla (Promega) using Lipofectamine 2000 (Invitrogen). Cells were treated with indicated inhibitor (10µM) with or without OA (5µg/mL) for 24H prior to collection and analysis using Promega Dual Luciferase assay kit per manufacturer’s specifications. Luciferase activity was measured using Veritas Luminometer (Promega); results are reported as relative bioluminescence units (BLU).

### Western blot analysis

Protein extraction and western blot analysis was performed as previously described [41]. Primary antibodies included SCD1 (Sigma-Aldrich, HPA012107), BiP (Cell Signaling, 3183), CHOP (Cell Signaling, 2895), CYP3A5 (Lifespan, ls-c96732), and β-actin (Sigma-Aldrich, A5441).

### Meta-analysis

Relationship between SCD1 expression and patient survival in different cancer subtypes was determined using KMPlotter, for gastric [42], lung [43], and ovarian [44] cancer. Affymetrix probe ID 200832_s_at was identified as optimal probe for SCD1 analysis using jetset [45], analysis was performed for relapse-free survival, patients split by median expression with best cutoff selected, no censoring for follow up threshold selected. For ccRCC meta-analysis, cBioPortal [46] was used to query the relationship between patient survival and SCD mRNA expression z-scores (RNA Seq V2 RSEM) using the dataset established by TCGA [47], where ∼5% of patients demonstrated upregulated SCD mRNA.

### IVIS bioluminescent imaging

ACHN cells were infected with pSIN Luc Ub Emerald GFP lentivirus construct, a kind gift from Dr. Yasuhiro Ikeda (Mayo Clinic). Lentivirus was prepared in 293FT viral progenitor cells (ATCC) with ViraPower™ using the transfectant Lipofectamine 2000 (Invitrogen) per the manufacturer protocol. Cells were sorted based upon GFP expression using BD FACSAria II flow cytometry cell sorter (BD Biosciences), yielding a 90% GFP-positive population. Bioluminescent imaging of *ex vivo* tissue bearing pSIN Luc Ub Emerald GFP expressing tumors was performed using IVIS® Spectrum (Perkin Elmer). D-Luciferin (GoldBio) was given at 30mg/mL, 0.1mL/mouse via intraperitoneal injection prior to lung tissue harvest and imaging. Lesion width was calculated as the mean of all nodules measured (7-12 per mouse), identified by H&E staining.

### *In vivo* tumor studies

A498 cells were injected subcutaneously in the flank of athymic nude mice at 1x10^6^/mouse. Once tumor burden reached >50mm^3^, SSI-4 treatment was administered at 60, 180, or 600 mg/kg in custom AIN76 animal chow (Research Diets, Inc.), given continuously for 4.5 weeks. Tumor burden was measured using calipers. For the lung tumor study, 2 million ACHN- pSIN Luc Ub Emerald GFP cells were injected intravenously in 50µL of PBS in athymic nude mice. 54 days after implantation, animals began SSI-4 treatment (180 mg/kg, chow, continuous) for 28 days. Once therapy was completed, lung tissue was harvested and analyzed for tumor burden via *ex vivo* bioluminescent imaging, and hematoxylin and eosin (H&E) staining of formalin-fixed, paraffin-embedded lung tissue. 20× images were obtained using Scanscope XT and Imagescope software. ACHN-bearing animals demonstrated a decreased rate of consumption, and corrected dosing was calculated to be 20mg/kg.

### *In vitro* evaluation of SCD1 inhibition by LC/MS

Microsomes were isolated from murine liver using Microsome Isolation Kit (BioVision) per the manufacturer protocol. Successful isolation of the microsome fraction was validated by enrichment of cytochrome P450 3A5 (CYP3A5), an endoplasmic reticulum resident protein, detected via western blot. Each assay (100 μL) contained 50 μg of microsomes, 1 μL of DMSO (for no drug controls) or DMSO-solubilized SCD1 inhibitor, 1 mM reduced NADH, 60 μM coenzyme A, 1 mM ATP, 1 mM DTT, and 5 mM MgCl_2_ in 100 mM sodium phosphate buffer (pH 7.4), analogous to earlier work [48]. Assays were incubated for five minutes at 25 °C and then supplemented with 30 µM ^13^C_18_-stearoyl CoA lithium salt (Sigma #675776) to initiate SCD1-catalyzed conversion to ^13^C_18_-oleoyl CoA. For each evaluated compound (i.e. SSI-1-4 and known inhibitor control MF-438), assays were conducted in duplicate at eight concentrations ranging from 0.125 nM to 500 nM. Following incubation at 37 °C for 30 min, enzymes were inactivated and precipitated by adding two volumes (200 µL) of acetonitrile and samples supplemented with 1 µM heptadecaonyl CoA (Sigma H1385) internal standard (I.S.) for LC/MS analyses. Samples were centrifuged at 13,000 rpm for 10 min. To quantify biotransformation of ^13^C_18_-stearoyl CoA to ^13^C_18_-oleoyl CoA for drug treatments relative to vehicle controls, supernatants were evaluated using the LC/MS^2^ acyl CoA quantitation method of Magnes et al. [49] and adapting selected reaction monitoring (SRM) for isotope-labeled acyl CoAs. Specifically, SRM was executed for ^13^C_18_-stearoyl CoA (*m/z* 1052 → 545), ^13^C_18_-oleoyl CoA (*m/z* 1050 → 543), and heptadecanoyl CoA I.S. (*m/z* 1020 → 513). LC/MS^2^ was conducted using a Thermo LTQ mass spectrometer interfaced with a Dionex UltiMate 8000 LC system. SRM signal areas for isotope-labeled acyl CoAs were measured relative to the heptadecanoyl CoA I.S. using XCalibur software (Thermo), and percent enzyme inhibition determined by comparing ^13^C_18_-oleoyl CoA quantities between treatment and DMSO control assays. Dose-response curves were prepared using GraphPad Prism.

### Preclinical pharmacokinetics

Pharmacokinetic assessment was performed for SSI-4 administered to fasted male C57BL/6 mice either intravenously (IV) or by oral gavage (PO) in DMSO:PEG400:water (10:70:20) as a clear solution at 5 mg/mL (IV dosing), and 1,3 or 10 mg/mL (respectively for the PO dosing). Serum analysis was performed by LC-MS-MS. PK calculation settings were obtained using the Phoenix WinNonlin 6.3 program, using either the Noncompartmental model 201 (IV bolus input) or Noncompartmental model 200 (extravascular input).

### Toxicology analysis

Sprague dawley rats (6-8 weeks old M/F) were orally gavaged with SS1-4 at the indicated dose daily, for a duration of seven days. For each dose levels 3 male and 3 females were placed on study. Rats were clinically observed and weighed daily. On day 8 rats were euthanatized and blood was collected by cardiac puncture. Blood chemistry was measured by STAT Veterinary laboratory (San Diego CA).

### Statistical analysis

ANOVA was used to determine the statistical significance of the differences between experimental groups, where p<0.05 as indicated by asterisk (*). Kaplan-Meier survival plots were generated using either KMPlotter or cBioPortal, with logrank P value calculated by host software.

## SUPPLEMENTARY MATERIALS FIGURES AND TABLES










