# Supplementary material for: Accelerated bottom-up drug design platform enables the discovery of novel stearoyl-CoA desaturase 1 inhibitors for cancer therapy
Source: Oncotarget. 2017 Oct 6;9(1):3–20. doi: 10.18632/oncotarget.21545 (PMC5787466; doi:10.18632/oncotarget.21545)
Supplement: Supplementary file 1 [file oncotarget-09-3-s001.pdf]

Accelerated bottom-up drug design platform enables the discovery of novel stearoyl-CoA desaturase 1 inhibitors for cancer therapy

SUPPLEMENTARY MATERIALS

Novel Ligand Generation

Using methodology of combining multiple data sources for an accelerated drug discovery process, multiple new compounds using core generation were generated. First, all the cores were separated ("core separation") from each known scaffold, leaving the binding features from the edges of each compound. Then, potential core fragments were combined from varied sources (core libraries, in-house fragment libraries, and de novo scaffold manipulations), which are then inserted back into the core slots ($Core_1$, $Core_2$, or $Core_3$). Each new core is fused with the existing chemistry on the edges and placed into the appropriate pool ($pool_1$, $pool_2$, or $pool_3$) (Figure S2). Each pool is filtered using energy minimization, correct bond orders, and ligand preparation with LigPrep. These three pools are then combined into a common de novo ligand pool. The entire pool of ligands is expanded to allow for generation of tautomers where appropriate, ionization states over a valid range of pH values, and isomerizations. Reactive functional groups are screened and removed from the dataset. At this point, Z-scoring filtering is applied as a reductive filter for SCD1 specificity.

Shape fitting algorithms

Shape similarity models were generated for each compound versus the known inhibitor, SAR707 or A939572, such that upon superposition of the known compound (A) and the de novo compound (B), the following measurement for jointly occupied volume $V_{A \cap B}$ is obtained, which is normalized by the total volume $V_{A \cup B}$, thus giving the normalized shape similarity Sim_{AB} ranging from 0 to 1. Thus, $Sim_{AB} = V_{A \cap B} / V_{A \cup B}$, where A is the known inhibitor SAR707 or A939572 and B is the unknown, de novo compound, were computed for 1000s of generated compounds. Finally, a pairwise method was employed for faster calculations (600 conformers per second) (Sastry, G.M. et al. *J. Chem. Inf. Model.* 2013;53:1531-1542; Pala, D. et al. *J. Chem. Inf. Model.* 2013;53(4):821-835; Kalid, O. et al. *J. Comput. Aided Mol. Des.* 2012;26:1217–1228; Fu, J. et al. *Bioorg. Med. Chem. Lett.* 2012;22(2):6848-6853; Sastry, G.M. et al. *J. Chem. Inf. Model.* 2011;51:2455-2466). Each compound is allowed to generate 500 conformers, retaining 20 conformers per rotatable bond, allowing the amide bonds to vary conformation, to maximize shape matching likelihood. Volume was also computed for both Pharmacophore types and Atom types using the Macromodel definition for atom typing. Four alignments per ligand were used, filtering out conformers with similarity below 0.7 and then compiled all the shape data together selecting the top shape matching compounds for retention and excluding all lower scoring hits. Various models for chemical shape can also be employed to screen both shape and chemical information simultaneously.

Computational docking

Initial docking was performed using Glide (v. 5.6) within the Schrödinger software suite (Schrödinger, LLC) (Mohamadi, F. et al. *J Comput Chem.* 1990;11(4):440-467). The origin of the compounds is described in *De Novo Library Design*. The starting conformation of ligands was obtained by the method of Polak-Ribière conjugate gradient (PRCG) energy minimization with the Optimized Potentials for Liquid Simulations (OPLS) 2005 force field (Jorgensen, WL et al. *J Am Chem Soc.* Mar 16 1988;110(6):1657-1666) for 5000 steps, or until the energy difference between subsequent structures was less than 0.001 kJ/mol-Å units. The docking methodology has been described previously (Caulfield, T. et al. *Proteins.* Nov 2012;80(11):2489-2500; Loving, K. et al. *Journal of computer-aided molecular design.* Aug 2009;23(8):541-554; Vivoli, M. et al. *Mol Pharmacol.* Mar 2012;81(3):440-454), and the scoring function utilized is described elsewhere (Friesner, RA et al. *Journal of medicinal chemistry.* oct 19 2006;49(21):6177-6196).

Molecular modeling of SCD1

Molecular models were generated for the human protein stearoyl-CoA desaturase 1, or acyl-CoA desaturase (SCD1) (NP_005054.3), which has the following 359 amino acid protein sequence: MPAHLLQDDISSSYTTTTTITAPPSRVLQNGGDKLETMPLYLEDDIRPDIKDDIYDPTYKDKEGPSPKVEYVWRNI ILMSLLHLGALYGITLIPTCKFYTWLWGVFYYFVSALGITAGAHRLWSHRSYKARLPLRLFLIIANTMAFQNDVYE WARDHRAHHKFSETHADPHNSRRGFFFSHVGWLLVRKHPAVKEKGSTLDLSDLEAEKLVMFQRRYYKPGLLM MCFILPTLVPWYFWGETFQNSVFVATFLRYA VVLNATWLVNSAAHLFGYRPYD KNISPRENILVSLGAVGEGFHN YHHSFPYDYSASEYRWHINFTTFFIDCMAALGLAY DRKKVSKAAILARIKRTGDGNYKSG. The modeling for this protein was completed using three combined methods, Schrodinger Prime modeling, TASSER, and Yasara structural and homology modeling program (Krieger E, Dunbrack RL, Jr., Hooft RW, Krieger B. Assignment of protonation states in proteins and ligands: combining pKa prediction with hydrogen bonding network optimization. *Methods Mol Biol.* 2012;819:405-421; Krieger E, Joo K, Lee J, et al. Improving physical realism, stereochemistry, and side-chain accuracy in homology modeling: Four approaches that performed well in CASP8. *Proteins.* 2009;77 Suppl 9:114-122; *Prime, version 2.1* [computer program]. Schrodinger, LLC, New York, NY 20092014; Zhou H, Skolnick J. Protein structure prediction by pro-Sp3-TASSER. *Biophys J.* Mar 18 2009;96(6):2119-2127; Zhou H, Skolnick J. Improving threading algorithms for remote homology modeling by combining fragment and template comparisons. *Proteins.* Jul 2010;78(9):2041-2048; Zhou H, Skolnick J. Template-based protein structure modeling using TASSER(VMT). *Proteins.* Sep 14 2011).

Several models were generated and compared for hybrid modeling, which resulted in best scoring portions from each program and validated for dihedrals, packing, and other metrics like Phi-Psi space.

From these final models for SCD1 were mapped using the SiteMap module to score highest confidence binding sites on SCD1, which resulted in a deep internal pocket that would correspond with lipophilic substrate binding, required for stearoyl-coA binding (*Schrödinger Suite 2014* [computer program]. BioLuminate, version 1.0. New York, NY: Schrödinger, LLC; 2014; Halgren T. New method for fast and accurate binding-site identification and analysis. *Chem. Biol. Drug Des.* 2007;69(146–148); Halgren T. Identifying and characterizing binding sites and assessing druggability. *J. Chem. Inf. Model.* 2009;49:377-389).

Prior to mapping out potential grid surfaces for an active site region surrounding residues, the ProteinPreparationWizard contained in Schrödinger (*Protein Preparation Wizard; Epik version 2.8; Impact version 6.3; Prime version 3.5* [computer program]: Schrödinger, LLC, New York, NY, USA; 2014) was used. The top regions identified using SiteMap region were then mapped for grid generation and decomposition of the protein's three-dimensional space for docking experiments. Using this grid, initial placement for A939572, SAR707, and other known inhibitors were docked using the Glide algorithm within the Schrodinger suite as a virtual screening workflow (VSW). The docking was accomplished using a scheme that proceeds from single-precision (SP) through extra-precision (XP) with the Glide algorithm (Glide, v. 5.7, Schrödinger, LLC). The top seeded poses were ranked for best scoring pose and unfavorable scoring poses were discarded. Each conformer was allowed multiple orientations in the site. Site hydroxyls, such as in serines and threonines, were allowed to move with rotational freedom. Docking scores were not retained as useful, since covalent bonding is the outcome. Hydrophobic patches were utilized within the virtual screening workflow (VSW) as an enhancement. Top favorable scores from initial dockings of yielded thousands of poses with the top five poses retained. XP descriptors were used to obtain atomic energy terms like hydrogen bond interaction, electrostatic interaction, hydrophobic enclosure and π-π stacking interaction that result during the docking run. VSW docking was completed for all novel generated compounds from the de novo design process described in the QSAR section and the docking utilized both hydrophobic constraints and the seeding generated from known inhibitor docking poses. Molecular modeling for importing and refining the known inhibitor compounds and generation of the small molecule compounds were prepared with LigPrep module (*LigPrep 2.2* [computer program]. Schrodinger, LLC, New York, NY, 2008: Schrödinger; 2010). All image rendering used for figures was completed with Maestro (*Maestro 9.4* [computer program]. New York, NY: Schrödinger, LLC; 2014).

QSAR Method

Active, Inactive, Unknown Ligand Preparation

Twenty active compounds that ranged from 3 nM to 400 nM and eight inactives ranging from 2.68 micromolar to >10 micromolar were built into our pharmacophore modeling system. Conformers were generated for all actives, inactives, and test set compounds using ConfGen and Mixed MCMM/LCMOD within Schrodinger (Watts KS, Dalal P, Murphy RB, Sherman W, Friesner RA, Shelley JC. ConfGen: A Conformational Search Method for Efficient Generation of Bioactive Conformers. *J.Chem. Inf. Model.* 2010;50:534-546). For ConfGen the number of conformers per rotatable bond was set at 100, maximum number of conformers per structure was set at 1000, sampling was set on "Thorough" mode and included preprocess

minimization of 100 steps and postprocess minimization of 50 steps and eliminate high-energy/redundant conformers. The MacroModel options for conformer generation used the OPLS2005 force field, GB/SA water solvation treatment and default setting for the maximum relative energy difference and maximum allowed atom deviation.

Pharmacophore Hypothesis Generation

A common pharmacophore was determined over a variant list that ranged from 5-6 sites and required a match from at least 6 of the 17 actives built into the model. The variant list included 187 selections based on sites created for all the actives chosen. The following letter code was used for the pharmacophore sites/features: hydrogen bond acceptor (A), hydrogen bond donor (D), hydrophobic group (H), negatively charged group (N), positively charged group [P], aromatic ring [R], and no custom features for X, Y, or Z designation were assigned. For images with pharmacophore models, the following appearance is given: (A) is light red sphere located at atom with lone pair and arrow point toward the lone pair, (D) is light blue sphere centered on hydrogen atom with arrow in direction of the potential H-bond, (H) is green sphere, (N) is red sphere, [P] is blue sphere, and [R] is orange torus in plane of aromatic ring. Our search method employed for finding common pharmacophores is identified using a tree-based partitioning technique that groups according to inter-site distances (k-points). Using a binary decision tree, a tree depth of five was allowed and partition into bins based on a 2.0 Å width, while partition continues to either eliminate or survive the procedure.

Variant motifs that were included were AAAAAA, AAAAAD, AAAAAH, AAAAAR, AAAADD, AAAADH, AAAADR, AAAAHH, …, HNPRRR, which resulted in the following top variant hypotheses AAAHHR, AAAHRR, AAHHRR with 45, 1, and 57 maximum hypotheses, respectively. The initial pharmacophore modeling considered >20,000 hypotheses. Actives were scored using vector and site filtering to keep RMSD below 1.200 Å, keep vectors with scores above 0.500, keep the top 30%, keep at least 10 and at most 50 using feature matching tolerances of A 1.00, D 1.00, H1.50, N 0.75, P 0.75, R 1.50, X 1.0, Y 1.0, and Z 1.0. The unweighted survival score formula is 1.0*(vector score) + 1.0*(site score) + 1.0*(volume score) + 1.0^(number of matches - 1), however to take advantage of existing active compounds, the adjusted scoring function is 1.0*(vector score) + 1.0*(site score) + 1.0*(volume score) - 0.001*(reference ligand(s) relative conformational energy) + 1.0^(number of matches - 1) - 0.001*(reference ligand activity).

This scoring metric resulted in eight qualified hypotheses for further examination, including: AAHHRR.2632, AAHHRR.2667, AAHHRR.2641, AAHHRR.2669, AAAHHR.5952, AAHHRR.2361, and AAAHHR.5952. All hypotheses had actives scored, inactives scored, then rescored for post-hoc analysis, which has the following formula: 1.0*(vector score) + 1.0* (site score) + 1.0*(volume score) + 1.0^(number of matches -1) - 0.001*(reference ligand relative conformational energy) + 0.001*(reference ligand activity). Then the "adjusted survival score" becomes the survival score - 1.0*(inactive match score).

The scoring hypotheses were bases on the identified pharmacophores from each surviving n-dimensional box for the chosen actives and additional information from partial matching of ligand alignments. The quality alignments were determined using three metrics, namely, the alignment score (via root-mean-squared-deviation (RMSD)), vector score (average cosine of the angles formed by corresponding pairs of vector features (A, D, R), and volume score (overlap of van der Waals models of non-hydrogen atoms in each pair of structures). As well, site scores for each alignment were computed to augment the alignment score with a cutoff Calign, which combined the site score, vector score, and volume score with separate weights to yield a combined alignment score for each non-reference pharmacophore that was aligned with reference. All pharmacophores within a box were treated as a reference and the highest one selected as a hypothesis during multi-ligand alignment optimization. The final scoring function (survival score) was: , where W's represented the weights and S's represented the scores. Inactives were penalized by adjusting their alignment score, such that, when an inactive matches only k out of n sites, an effective n-point alignment score was computed as follows: $S_{align,n} = \sqrt{W_k S_{align,k}^2 + (1 - W_k) C_{align}^2}$, where W_k=k/n. The final adjusted score mentioned above became $S_{adjusted} = S_{actives} - W_{inactives} S_{inactives}$. Default weights were used with all equations, as shown above. Hypotheses were clustered for to tease out pharmacophore model variants with similar scores.

Generation of 3D QSAR Models

The 3D QSAR models were built by mapping the chemical features of ligand structures onto a cubic three-dimensional grid space with the smallest grid spacing of 1 Å per side. As above, the ligands are first aligned to the set of pharmacophore features for the selected hypothesis using a standard least-squares approach, which utilizes regression of the independent variables with binary-valued bits in the cubes by structural components and the dependent variables are the activities. The regression was performed via a partial least squares (PLS) method, where a series of models generated with increasing number

of PLS factors. T-value filter (t-value < 2.0) was used to eliminate independent variables overly sensitive to incremental changes from the training set. For structural components, both atom-based and pharmacophore features based were examined. The regression with m PLS factors, fitted to activities is given as:

$$\hat{y} = \mu^y + \sum_{i=1}^{m} b_i t_i$$

, where m = number of PLS factors, b is regression coefficient, vector y represents activity values in the training set. For the prediction of activities for the new ligands, the following was used:

$$\hat{y} = \mu^y + \sum_{i=1}^{m} (X_{k,i} - \mu_i^x) b_i^x$$

, where $Z_{scr} = \frac{(Shape_{norm} + Dock_{norm} + QSAR_{norm})}{3}$. Models with high stability were preferred.

Z-score matrix

A final Z-filtering mechanism was applied to reduce the dataset to a few select compounds for synthesis and experimental screening by using combined normalized scoring. The final Z-score for each compound was determined as:

$$Z_{scr} = \frac{(Shape_{norm} + Dock_{norm} + QSAR_{norm})}{3}$$

, which averages average of the sums the normalization of each individual Shape, the Dock, and 3D-QSAR score

Kinome Scan

Kinome scan performed as described by manufacturer http://services.discoverx.com/

SUPPLEMENTARY REFERENCES

1. Copland JA, Marlow LA, Kurakata S, Fujiwara K, Wong AK, Kreinest PA, Williams SF, Haugen BR, Klopper JP, Smallridge RC. Novel high-affinity PPARgamma agonist alone and in combination with paclitaxel inhibits human anaplastic thyroid carcinoma tumor growth via p21WAF1/CIP1. Oncogene. 2006; 25:2304–17.
2. Szász AM, Lánczky A, Nagy Á, Förster S, Hark K, Green JE, Boussioutas A, Busuttil R, Szabó A, Győrffy B. Cross-validation of survival associated biomarkers in gastric cancer using transcriptomic data of 1,065 patients. Oncotarget. 2016; 7:49322–33. https://doi.org/10.18632/oncotarget.10337
3. Győrffy B, Surowiak P, Budczies J, Lánczky A. Online survival analysis software to assess the prognostic value of biomarkers using transcriptomic data in non-small-cell lung cancer. PLoS One. 2013; 8:e82241.
4. Gyorffy B, Lánczky A, Szállási Z. Implementing an online tool for genome-wide validation of survival-associated biomarkers in ovarian-cancer using microarray data from 1287 patients. Endocr Relat Cancer. 2012; 19:197–208.
5. Li Q, Birkbak NJ, Gyorffy B, Szallasi Z, Eklund AC. Jetset: selecting the optimal microarray probe set to represent a gene. BMC Bioinformatics. 2011; 12:474.
6. Gao J, Aksoy BA, Dogrusoz U, Dresdner G, Gross B, Sumer SO, Sun Y, Jacobsen A, Sinha R, Larsson E, Cerami E, Sander C, Schultz N. Integrative analysis of complex cancer genomics and clinical profiles using the cBioPortal. Sci Signal. 2013; 6:pl1.
7. Creighton CJ, Morgan M, Gunaratne PH, Wheeler DA, Gibbs RA, Gordon Robertson A, Chu A, Beroukhim R, Cibulskis K, Signoretti S, Vandin Hsin-Ta Wu F, Raphael BJ, Verhaak RG, et al, and Cancer Genome Atlas Research Network. Comprehensive molecular characterization of clear cell renal cell carcinoma. Nature. 2013; 499:43–49.
8. Soulard P, McLaughlin M, Stevens J, Connolly B, Coli R, Wang L, Moore J, Kuo MS, LaMarr WA, Ozbal CC, Bhat BG. Development of a high-throughput screening assay for stearoyl-CoA desaturase using rat liver microsomes, deuterium labeled stearoyl-CoA and mass spectrometry. Anal Chim Acta. 2008; 627:105–11.
9. Magnes C, Sinner FM, Regittnig W, Pieber TR. LC/MS/MS method for quantitative determination of long-chain fatty acyl-CoAs. Anal Chem. 2005; 77:2889–94.

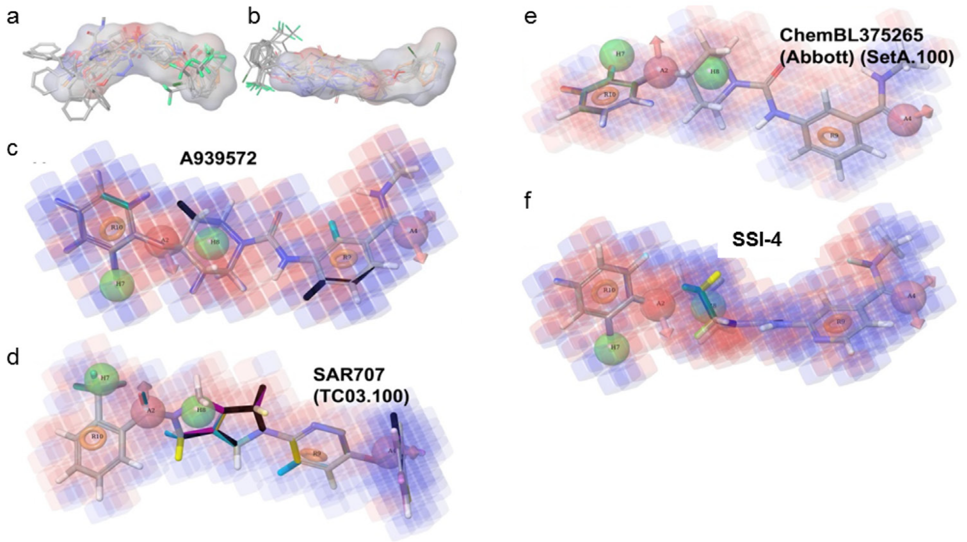

Supplementary Figure 1: QSAR modeling of top hits. (a) Shape fit of the top inhibitors with A939572 shape model given (surface rendered). (b) Improved shape fit for top inhibitors is shown for the SAR707 shape model (surface rendered). (c) QSAR fit for the compound A939572 with the top pharmacophore hypothesis. (d) QSAR fit for the compound SAR707 with the top pharmacophore hypothesis. (e) Improved QSAR fit for the compound ChemBL375265 with the top pharmacophore hypothesis. (f) Improved QSAR fit for the compound SetA.04 with the top pharmacophore hypothesis.

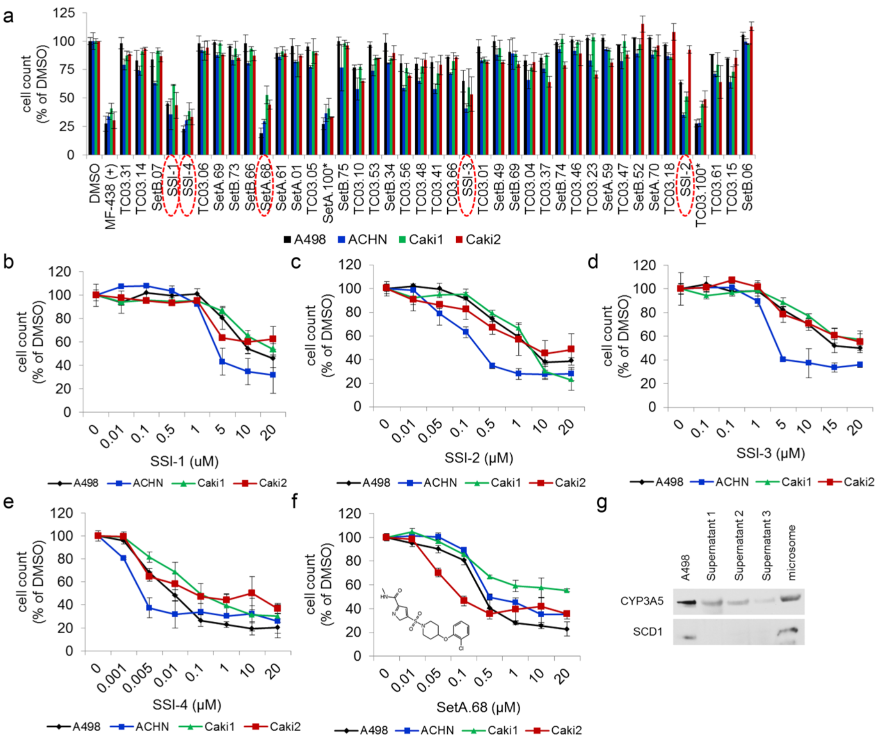

Supplementary Figure 2: IC$_{50}$ determination for SSI-1, SSI-2, SSI-3, and SSI-4. (a) High-throughput proliferative screen of known and novel SCD1 inhibitors in ccRCC cell lines. Dashed line represents a 40% decrease in proliferation as compared to DMSO control. Experimental compounds that achieved 40% or greater growth inhibition in a minimum of 2/4 cell lines are circled. MF-438 was included as a known SCD1 inhibitor. Compounds labeled with an asterisk (*) are single-blinded known SCD1 inhibitors. (b-f) Dose-response of SSI-1, SSI-2, SSI-3, SSI-4, and SetA.68 in four ccRCC cell lines. (f) The chemical structure for SetA.68 is embedded within the proliferative dose-response line graph. (g) Results of microsome isolation from mouse liver, demonstrating enrichment of resident ER protein CYP3A5 in the microsome fraction by western blot analysis. SCD1 protein expression in microsomes was examined; total A498 ccRCC cell lysate was used as a positive control.

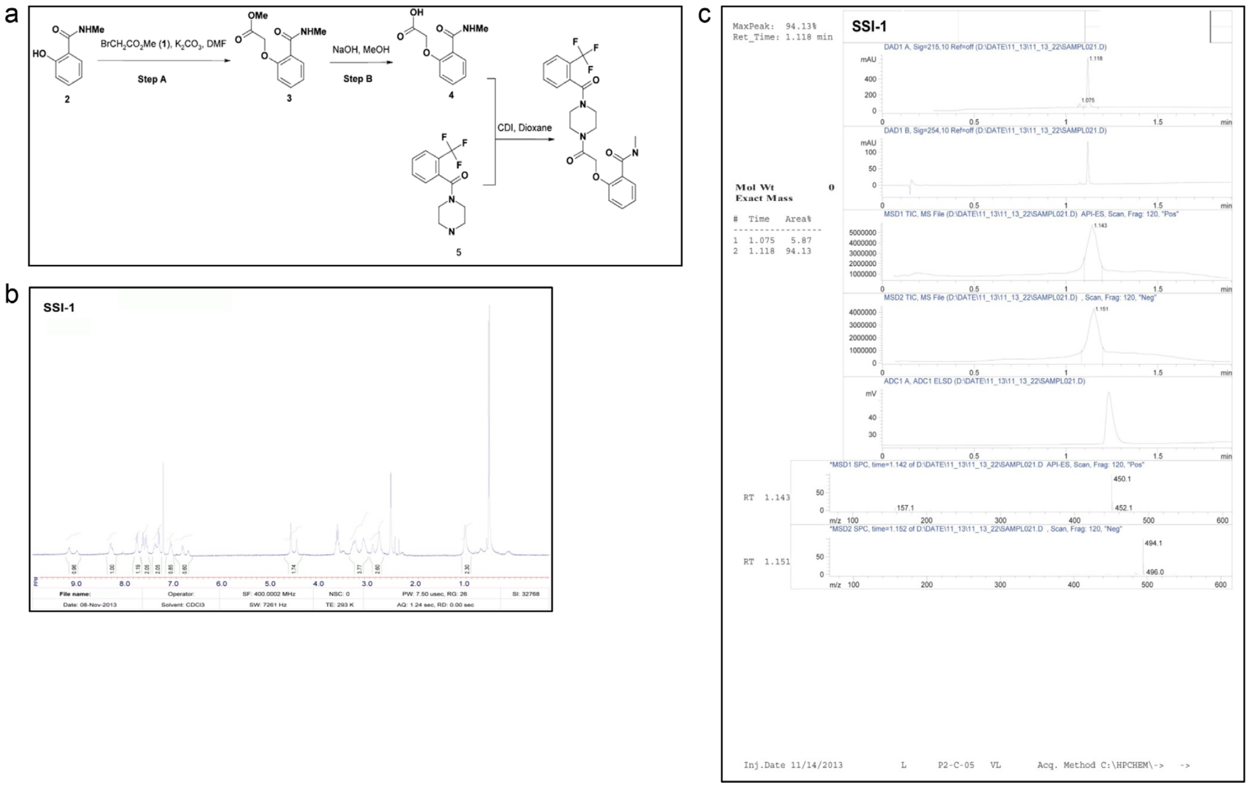

Supplementary Figure 3: Spectral information for SSI-1. (a) Synthesis route for SSI-1 was performed as follows: Step A- Potassium carbonate (2.76 g, 20 mmol) was added to a mixture of methyl bromoacetate (compound 1, 1.68 g, 11 mmol) and compound 2 (1.51 g, 0.01 mol) in DMF (50 mL) under vigorous stirring. The reaction mixture was stirred at r.t. for 24 h and then treated with water (200 mL) and extracted by CH_2Cl_2 (3×30 mL). The organic layer was washed with brine and dried under Na_2SO_4. The solvent was removed in vacuum giving compound 3 which was used for the next step without purification. Yield: 1.56 g (70%). Step B- Compound 3 (1.56 g, 7 mmol) in MeOH (20 mL) of was added to 10% NaOH (5 mL). The reaction mixture was stirred at r.t for 8 h and then treated with 5% HCl until pH ~ 3. The precipitate formed was filtered, dried and used for the next step without purification. Yield: 1.33 g (91%). Step C- CDI (117 mg, 0.72 mmol) was added to a solution of compound 4 (125 mg, 0.6 mmol) in dioxane (15 mL) under stirring and the mixture was heated at 50 °C for 30 minutes until end of liberation of gas. Then compound 5 (155 mg, 0.6 mmol) was added and the mixture was left stirring at 60 °C for 2 days (reaction is monitored by TLC). When reaction completed the mixture was treated with water (150 mL), extracted by CH_2Cl_2 (3×30 mL) washed with 5% Na_2CO_3 (30 mL), water (30 mL) and brine (30 mL), dried under Na_2SO_4. Then the solvent was removed in vacuum and the obtained residue was purified by flash chromatography to afford SSI-1. Yield: 135 mg (50%). (b) ^{1}H NMR and (c) LC/MS data for SSI-1 are shown herein in their entirety.

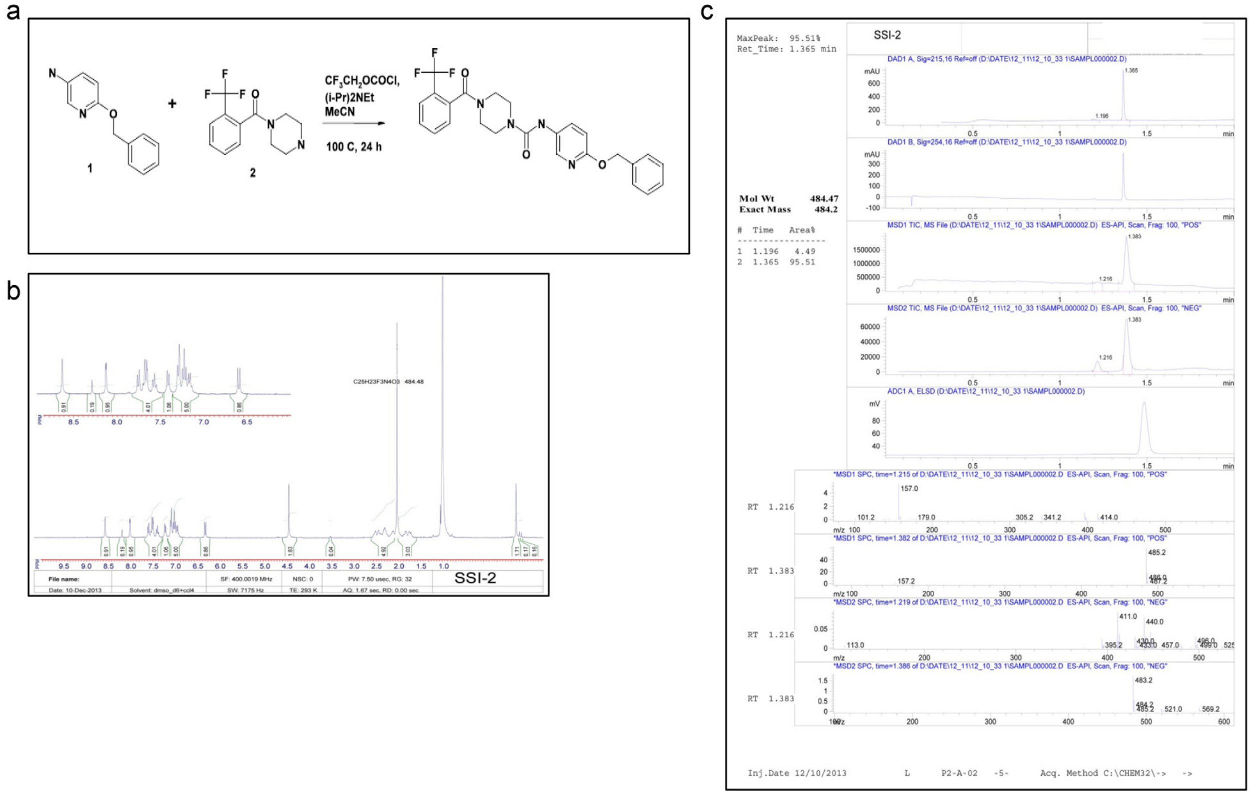

Supplementary Figure 4: Spectral information for SSI-2. (a) Synthesis route for SSI-2 was performed as follows: To a solution of compound 1 (200 mg, 1 mmol) in CH_3CN (20 mL) trifluoroethyl chlorocarbonate (240 mg, 1.2 mmol) and DIPEA (322 mg, 2.5 mmol) were added under stirring at r.t. After 30 min of stirring amine 2 (258 mg, 1 mmol) the reaction mixture was heated at 100 °C for 24 h, then cooled, treated with water (250 mL) and extracted by CH_2Cl_2 (3×30 mL), washed with water (30 mL) and brine (30 mL), dried under Na_2SO_4. Then the solvent was removed in vacuum and the obtained residue was purified by flash chromatography giving pure SSI-2. Yield: 266 mg (50%). (b) ^{1}H NMR and (c) LC/MS data for SSI-2 are shown herein in their entirety.

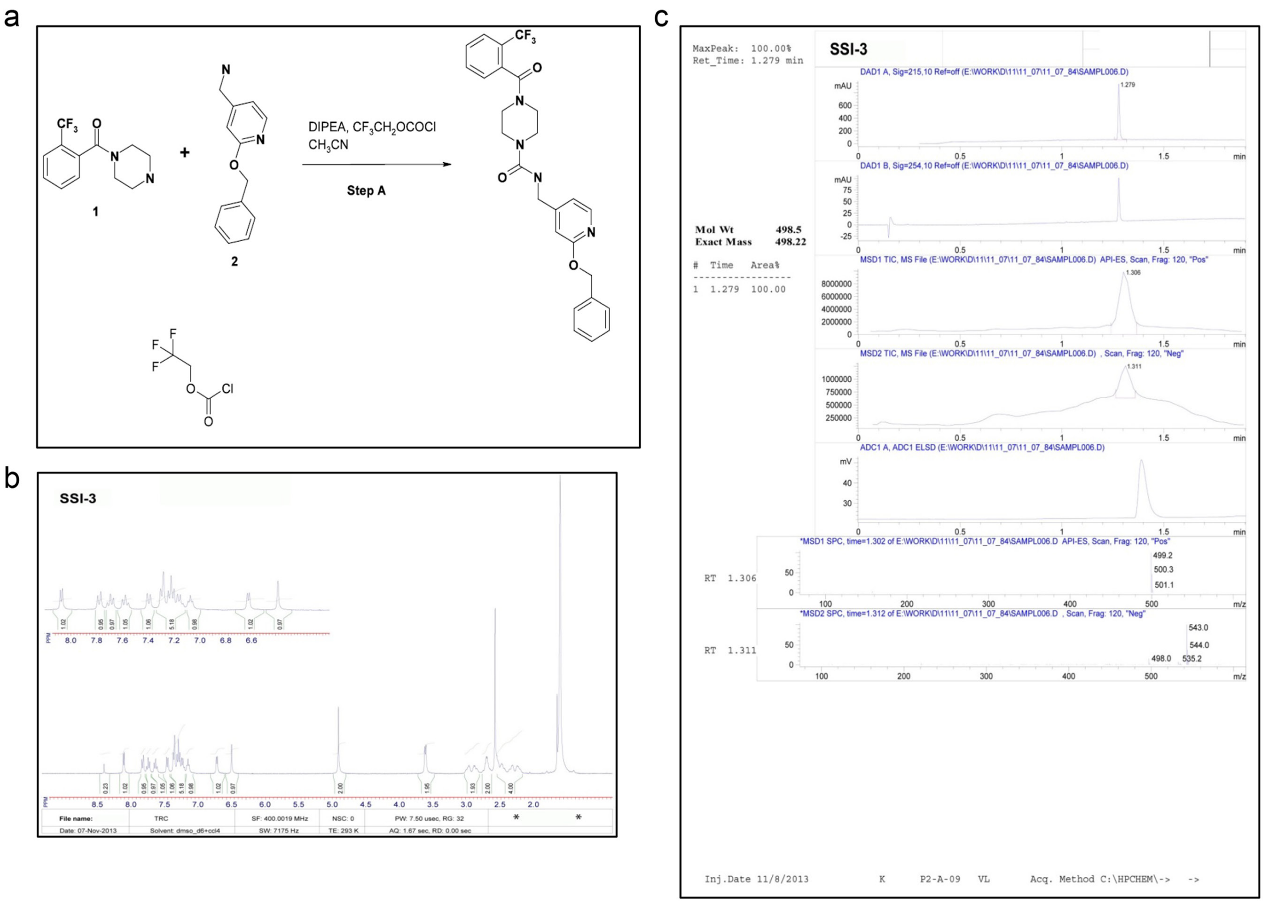

Supplementary Figure 5: Spectral information for SSI-3. (a) Synthesis route for SSI-3 was performed as follows: To a solution of compound 2 (214 mg, 1 mmol) in CH_3CN (30 mL) trifluoroethyl chloroformate (195 mg, 12 mmol) was added under stirring. Then diisopropyl ethyl amine (150 g, 2.5 mmol) and amine 3 (258 mg, 1 mmol). The reaction mixture was stirred at 100 C for 24 h. Then the mixture was treated with water (100 mL) and extracted by CH_2Cl_2 (3×30 mL). The organic layer was washed with water (30 mL) and brine (30 mL) and dried under Na_2SO_4. The solvent was removed in vacuum and the residue was purified by flash chromatography to give SSI-3. Yield: 314 mg (63 %). (b) ^{1}H NMR and (c) LC/MS data for SSI-3 are shown herein in their entirety.

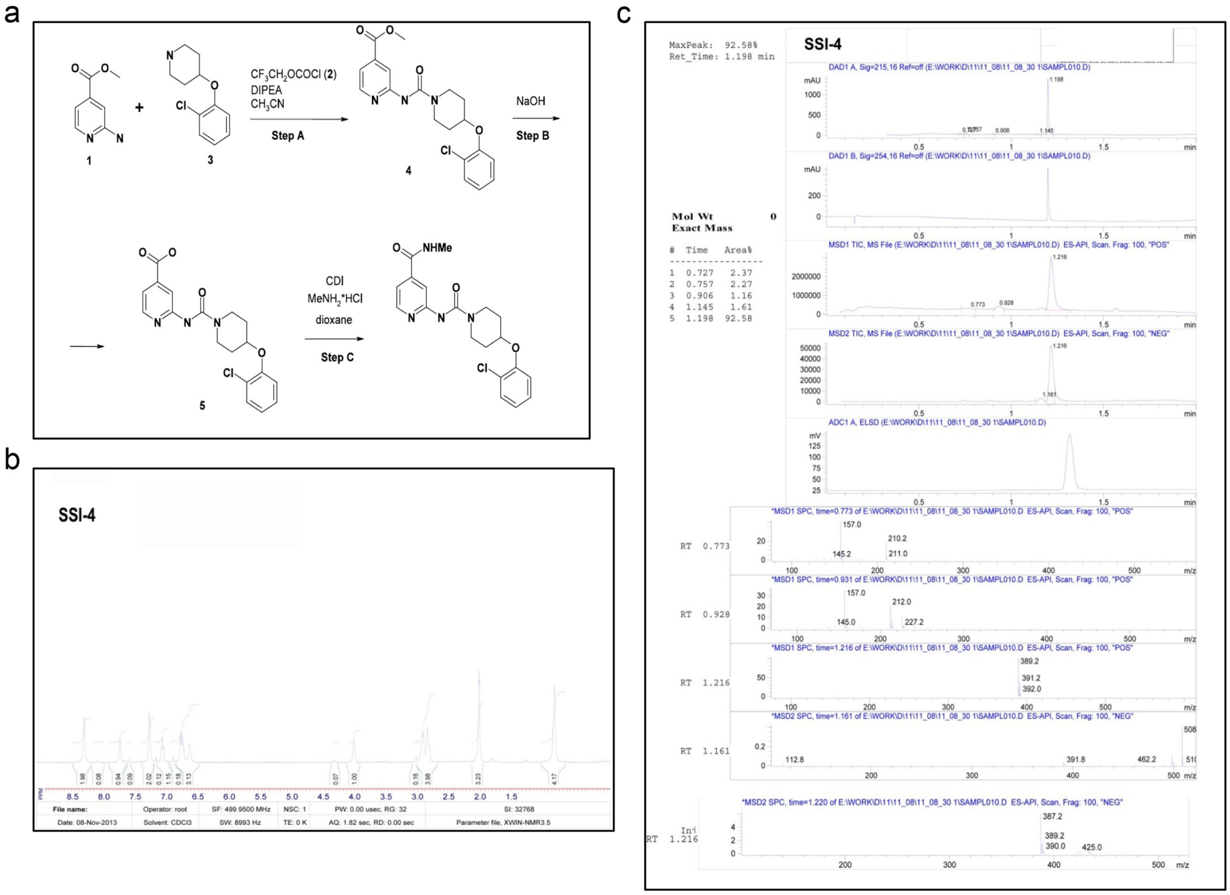

Supplementary Figure 6: Spectral information for SSI-4. (a) Synthesis route for SSI-4 was performed as follows: Step A- To a solution of compound 1 (1.52 g, 10 mmol) in CH_3CN (30 mL), compound 2 (1.95 g, 12 mmol) was added under stirring. Then diisopropyl ethyl amine (1.5 g, 25 mmol) and amine 3 (2.11 g, 10 mmol). The reaction mixture was stirred at 100 °C for 24 h. Then the mixture was treated with water (150 mL) and extracted by CH_2Cl_2 (3×30 mL). The organic layer was washed with water (30 mL) and brine (30 mL) and dried under Na_2SO_4. The solvent was removed in vacuum giving product 4 which was used for the next step without purification. Yield: 3.12 g (80%). Step B- Compound 4 (2.73 g, 7 mmol) in MeOH (20 mL) of was added to 10% NaOH (5 mL). The reaction mixture was stirred at r.t for 8 h and then treated with 5% HCl until pH ~ 3. The precipitate formed was filtered, dried giving compound 5 which was used for the next step without purification. Yield: 2.24 g (85%). Step C- To a solution of compound 5 (376 mg, 1 mmol) in dioxane (20 mL) CDI (194 mg, 1.2 mmol) was added and heated at 50 °C for 30 min until completion of gas liberation. Then methyl amine hydrochloride (96 mg, 3 mmol) was added and the mixture was stirred at 60 C for 2 h. Then the reaction mixture was cooled and treated with water (100 mL) and extracted by CH_2Cl_2. The organic layer was washed with 10% Na_2CO_3 (20 mL), brine (20 mL) and dried under Na_2SO_4. The solvent was removed in vacuum and the residue was purified by flash chromatography to afford SSI-4. Yield: 148 mg (38%). (b) ^{1}H NMR and (c) LC/MS data for SSI-4 are shown herein in their entirety.

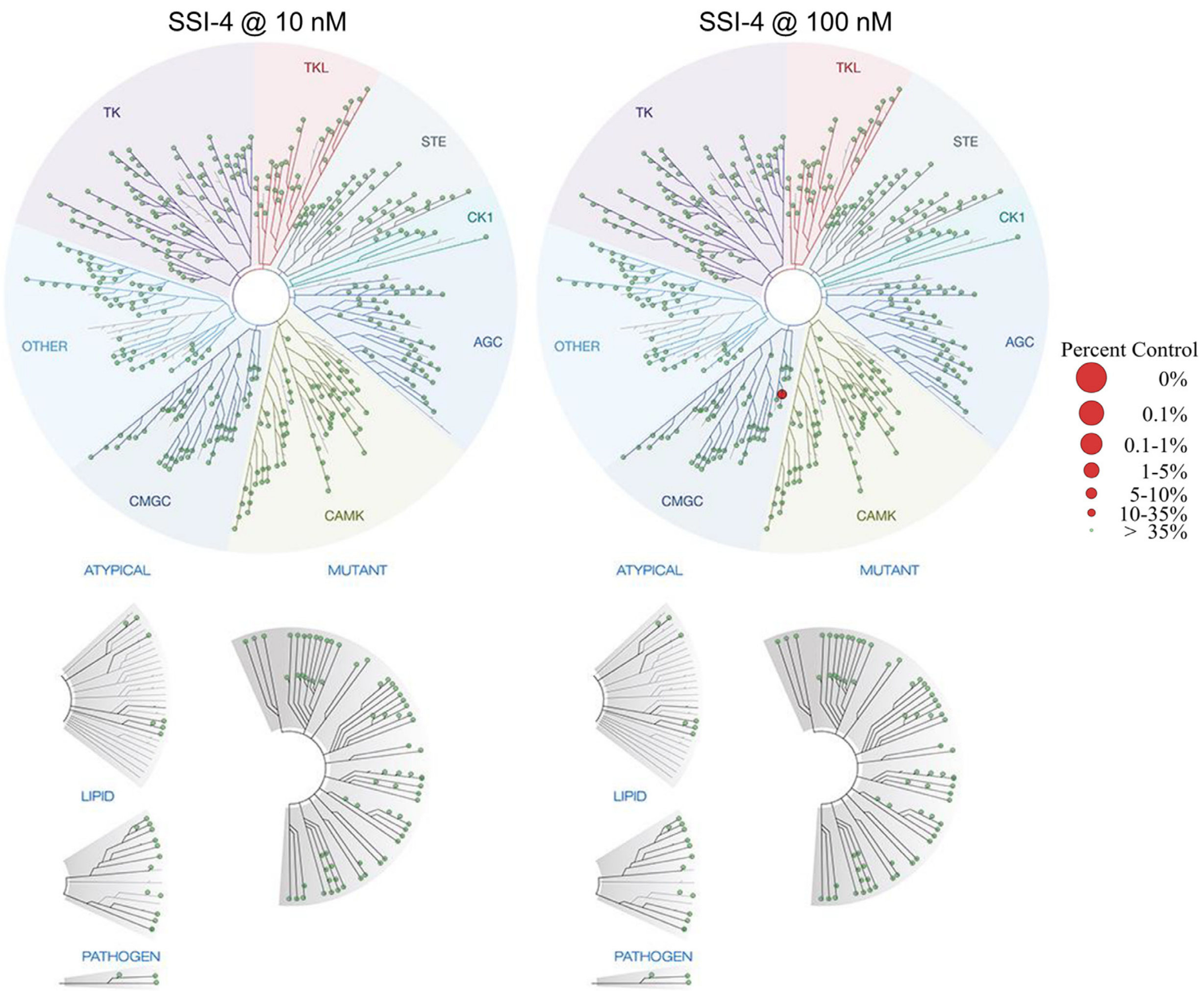

Supplementary Figure 7: Results of SSI-4 kinome scan shown as TREE*spot*™ chart, where kinases found to bind are marked with red circles. Larger circles indicate higher-affinity binding.

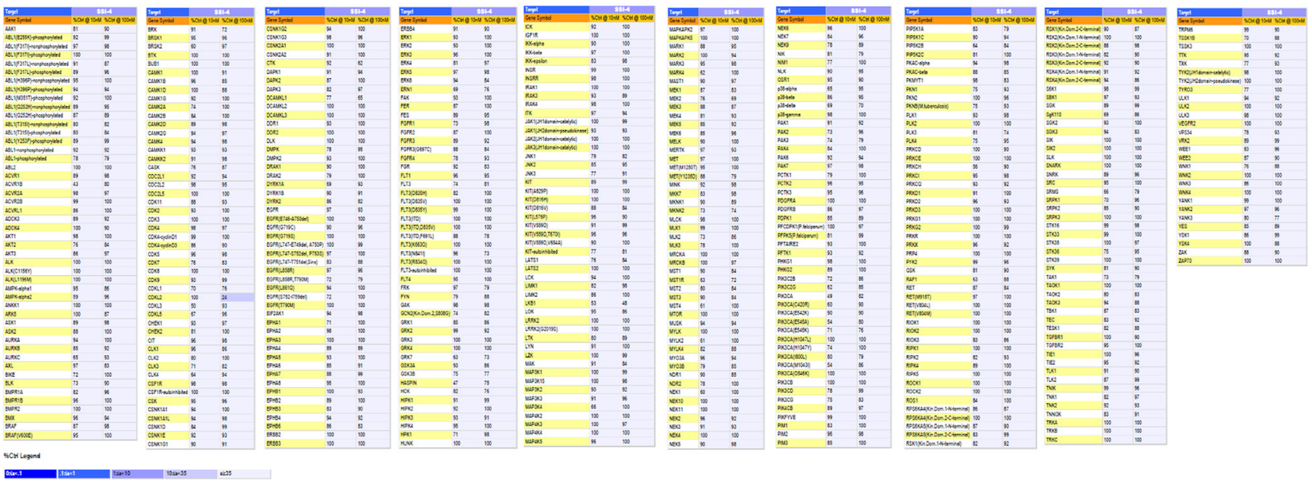

Supplementary Figure 8: SSI-4 kinome scan, full matrix. SSI-4 was screened at 10 nM and 100 nM for binding to a total of 468 kinases. Results for primary screen binding interactions are reported as % control, where lower numbers indicate stronger hits in the matrix.

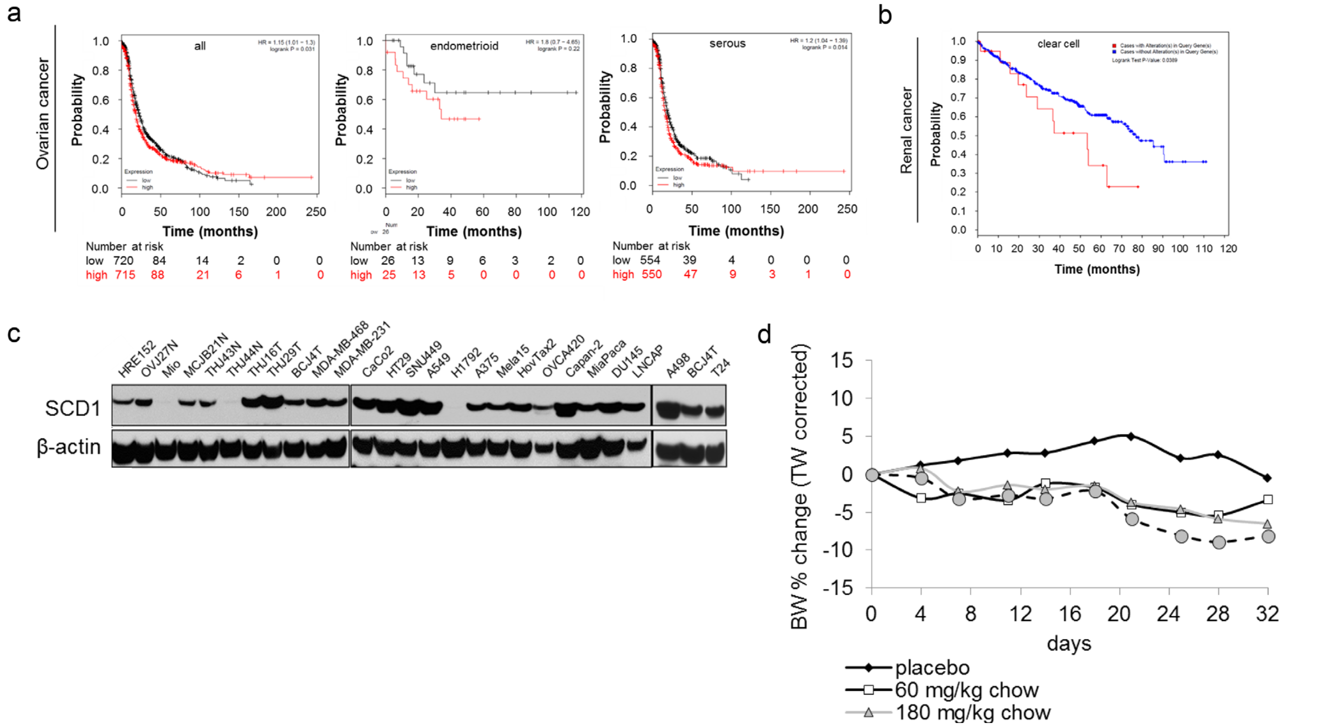

Supplementary Figure 9: Patient survival correlated with SCD1 gene expression. (a) Kaplan Meier survival analysis of indicated subtypes of ovarian cancer patients sorted into high versus low SCD1 mRNA expression. Patient risk assessment at indicated mean follow-up is shown. (b) Kaplan Meier survival analysis in clear cell renal cell carcinoma patients, sorted by SCD1 mRNA upregulation which occurred in 24/499 patients. (c) Western blot showing relative SCD1 protein expression that correspond to tumor cell lines used in the high-throughput proliferative screen (Fig. 3d). β-actin is included as a loading control. (d) Change in body weight (BW) corrected for individual total weight, recorded during *in vivo* dose-response to SSI-4 in A498 xenograft model (Figure 3g).

For Supplementary Tables see in Supplementary Files.
